# Conformational gating, dynamics and allostery in human monoacylglycerol lipase

**DOI:** 10.1038/s41598-020-75497-5

**Published:** 2020-10-28

**Authors:** Sergiy Tyukhtenko, Xiaoyu Ma, Girija Rajarshi, Ioannis Karageorgos, Kyle W. Anderson, Jeffrey W. Hudgens, Jason J. Guo, Mahmoud L. Nasr, Nikolai Zvonok, Kiran Vemuri, Gerhard Wagner, Alexandros Makriyannis

**Affiliations:** 1 Center for Drug Discovery and Departments of Pharmaceutical Sciences and Chemistry and Chemical Biology, Northeastern University, Boston, MA 02115-5000 USA; 2 BioProcess Measurements Group, Biomolecular Measurement Division, National Institute of Standards & Technology, Rockville, MD 20850 USA; 3 Institute for Bioscience and Biotechnology Research, 9600 Gudelsky Drive, Rockville, MD 20850 USA; 4 Barnett Institute for Chemical and Biological Analysis, Northeastern University, Boston, MA 02115-5000 USA; 5 Department of Biological Chemistry and Molecular Pharmacology, Harvard Medical School, Boston, MA 02115 USA; 6 Renal Division and Division of Engineering in Medicine, Department of Medicine, Brigham and Women’s Hospital, Harvard Medical School, Boston, MA 02115 USA

**Keywords:** Enzyme mechanisms, Molecular conformation, Biochemistry, Biophysics, Chemical biology, Drug discovery, Structural biology

## Abstract

Inhibition of human Monoacylglycerol Lipase (hMGL) offers a novel approach for treating neurological diseases. The design of inhibitors, targeting active-inactive conformational transitions of the enzyme, can be aided by understanding the interplay between structure and dynamics. Here, we report the effects of mutations within the catalytic triad on structure, conformational gating and dynamics of hMGL by combining kinetics, NMR, and HDX-MS data with metadynamics simulations. We found that point mutations alter delicate conformational equilibria between active and inactive states. HDX-MS reveals regions of the hMGL that become substantially more dynamic upon substitution of catalytic acid Asp-239 by alanine. These regions, located far from the catalytic triad, include not only loops but also rigid α-helixes and β-strands, suggesting their involvement in allosteric regulation as channels for long-range signal transmission. The results identify the existence of a preorganized global communication network comprising of tertiary (residue-residue contacts) and quaternary (rigid-body contacts) networks that mediate robust, rapid intraprotein signal transmission. Catalytic Asp-239 controls hMGL allosteric communications and may be considered as an essential residue for the integration and transmission of information to enzymes’ remote regions, in addition to its well-known role to facilitate Ser-122 activation. Our findings may assist in the identification of new druggable sites in hMGL.

## Introduction

Human monoacylglycerol lipase inhibitors have shown immense promise in treating a variety of human diseases^1–12^. Specifically, hMGL inhibitors have shown efficacy in animal models of traumatic brain injury^13^ and chronic traumatic encephalopathy (CTE)^6^, opioid dependence^4^, depression^7^, migraine^14^, anticipatory nausea^9^, neuropathic pain^1^, liver fibrosis^12^ and cancer^15–17^, neuroinflammation and neurodegeneration related disorders including Alzheimer’s disease^18–22^, Parkinson’s disease^23–27^ and multiple sclerosis^5^, and in humans, for treating Tourette’s syndrome^28^. Although known hMGL crystal structures (wild type open form^29,30^, PDB: 3HJU and 3JW8; closed form^3^, PDB: 3PE6) provide invaluable structural information for the design of inhibitors, static information alone is not enough to understand the conformational dynamics of the active site and its interactions with inhibitors. Rational design of novel hMGL inhibitors that target the conformational transitions and affect population shifts between open and closed forms requires knowledge of both the static structure and the dynamics of the enzyme.

**Figure 1:**
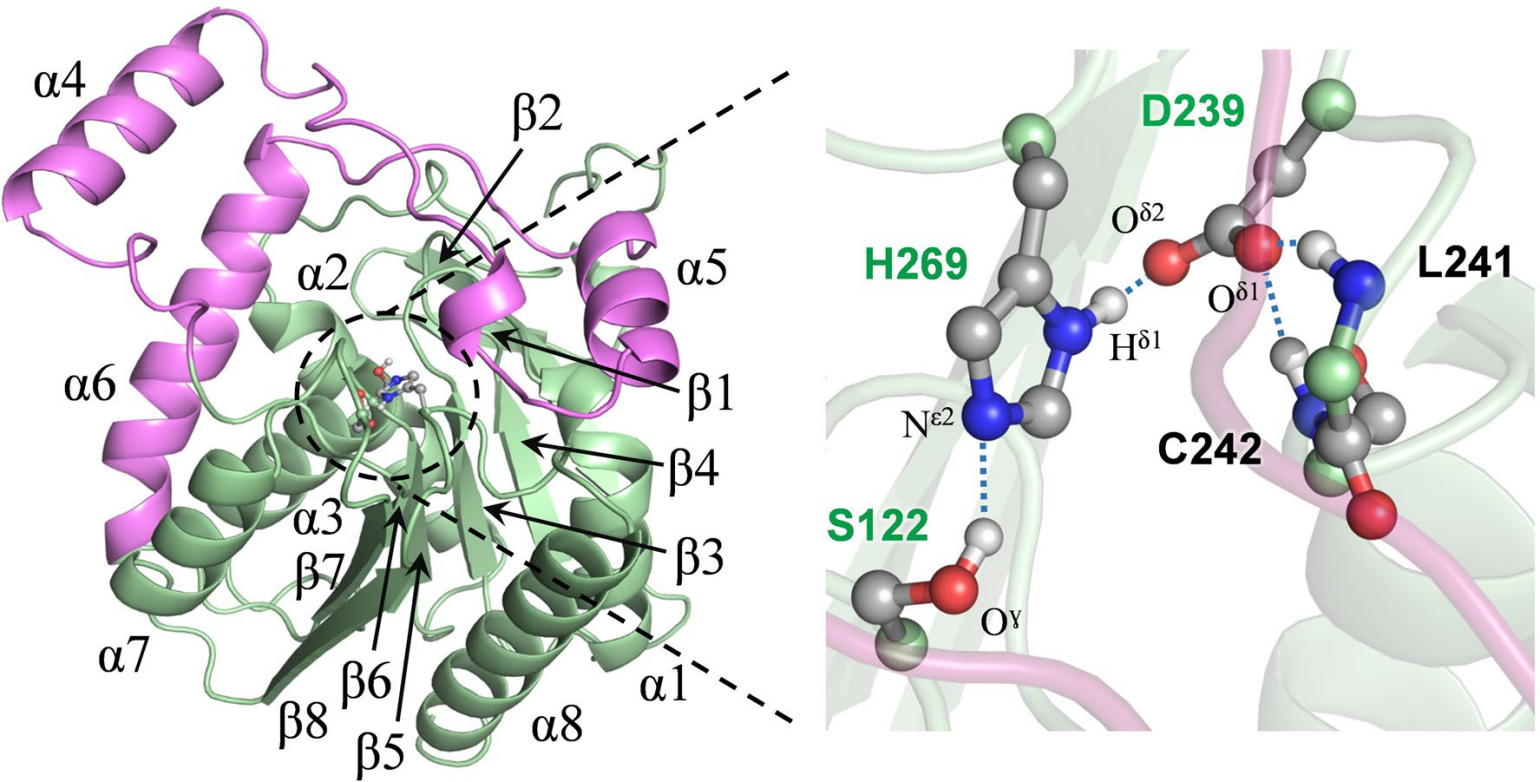
Position of the catalytic triad in the crystal structure of *apo*-hMGL (PDB 3HJU). The lid domain is highlighted in magenta (left). Close-up of the catalytic site (right) encompassing the catalytic triad Asp-239, His-269, Ser-122 with hydrogen bonded proximal residues. Fast exchanging H^δ1^ proton of the catalytic triad base His-269 is detectable by conventional NMR spectroscopy experiments.

hMGL is a serine alpha/beta-hydrolase (ABH) that cleaves the ester bond of 2-arachidonoylglycerol (2-AG) releasing arachidonic acid and glycerol. The catalytic triad (Ser-122, His-269 and Asp-239) is located at the bottom of the long channel under the lid domain of the enzyme (Fig. 1). The nucleophilic Ser-122 resides at the sharp turn between strand β5 and helix α3, a nucleophilic elbow with pronounced conformational rigidity. The basic residue His-269 resides within the loop between strand β8 and helix α8 and the catalytic acid Asp-239 is located at the turn after strand β7. Analysis of available hMGL crystal structures suggests that a highly preorganized collection of hydrogen bonds within the catalytic triad may be responsible for optimal positioning of all catalytically important residues (Fig. 1). In addition to the catalytic triad residues, this network also involves Leu-241 and Cys-242, buried in the active site close to the catalytic aspartic acid in the space and sequence. As a result, Asp-239 is an acceptor of three relatively strong hydrogen bonds, wherein one bond is formed between O^δ2^ of Asp-239 and H^δ1^ of His-269, and two hydrogen bonds exist between backbone amide protons of Leu-241 and Cys-242 and O^δ1^ of Asp-239. Additionally, several aromatic residues within the active site of hMGL could be involved in the structural stabilization of active site (see below). Site-directed mutagenesis of serine proteases demonstrates that alteration of any component of a catalytic triad results in a significant decrease in enzyme efficiency^31,32^.

Analysis of alpha–beta hydrolase active sites from 51 representative members of 40 structural ABH fold families led to a strong conclusion that the catalytic acid is usually surrounded by a planar, supporting scaffold^33,34^. This scaffold, together with the acid, forms the so-called catalytic acid zone, which coordinates the position of the catalytic histidine group and can compensate for the loss of function upon mutation of catalytic acid. Conserved substructures were identified around the nucleophile elbow and the oxyanion hole in selected representative structures from 40 ABH families^34^. In addition, a residue cluster comprising aromatic side chains that structurally stabilize the catalytic nucleophile and the oxyanion hole was identified. In our previous work we demonstrated the importance of the active site aromatic cluster involving His-272, Tyr-58 and His-269 for the stabilization of open (active) hMGL conformers^35^. Thus, the catalytic acid, nucleophile, and oxyanion zones, together with an aromatic cluster, coordinate the catalytic triad residues into proper positions for effective catalysis. Analyses of available hMGL crystal structures suggest that the components of the catalytic triad are highly connected, structurally stable residues that can serve as hub residues for rapid and robust propagation of allosteric effects. Potentially, these active site residues are critical for the transmission of information between residues in the protein^36^.

In a previous study, we established that solution NMR can resolve the open and closed conformational states of hMGL^35^. These states are defined by the position of the lid domain (residues 151–225), comprising helices α4, α5, α6, and two prolonged connecting loops. Transitions between open and closed states primarily involve large conformational movements of the highly flexible helix α4 and loops^3^. As a highly dynamic structure, the lid domain acts as a gate and plays a key role in hMGL function by controlling substrate access to the binding pocket. Residues at this gate and the residues at the active site may adopt both open- and closed- type configurations, indicating their potential involvement as anchoring residues responsible for reversible switching. The synchronized movements of these elements may provide an energetically favorable mechanism for controlling selectivity and substrate accessibility to the binding pocket of hMGL.

The molecular details of allosteric communications between the active site and the distal regions are still poorly understood. We have shown previously that the native state of hMGL is a conformational ensemble and that introducing an allosteric perturbation by single mutation of a critical distal residue can significantly affect the networks of allosteric interactions, resulting in conformational rearrangements in the active site and other regions of hMGL^37^.

This study investigates the role of the catalytic triad and neighboring residues that mediate conformational gating and allostery in hMGL. In particular, this investigation explores the consequences of breaking the Asp-239–His-269 hydrogen bond in the catalytic triad. Herein we combine and analyze enzyme kinetics data, NMR, CD and hydrogen–deuterium exchange mass spectrometry (HDX-MS) measurements, along with metadynamics simulations, to investigate the structural, dynamical, and functional responses to mutations within the catalytic triad of hMGL.

## Results

### Active site residues selected for mutations

Using doubly mutated **(**L169S/L176S), soluble hMGL (sol-hMGL) as a template that supports NMR experiments^35,37^ we constructed mutants containing either conservative or nonconservative substitutions of the active site residues: S122A, S122T, S122C, H269A, D239A, D239N, L241A, C242A, H121A and Y268A. CD and NMR spectroscopy measurements (Figs. S1–S3) indicated that these mutants retain proper secondary and tertiary folds (see Supplementary Information (SI) for details).

### Impact of active site substitutions on steady state kinetics of hMGL

We measured the enzyme kinetics of each hMGL construct with the endogenous substrate, 2-AG. Fig. S4 exhibits the Michaelis–Menten plots (saturation plots) observed for all designed constructs. Table 1 displays the derived steady-state kinetic parameters. Nonconservative substitutions of Ser-122 nucleophile and His-269 catalytic base residues for alanine induce complete loss of enzymatic activity. Conservative substitutions of Ser-122 for cysteine and threonine exhibit activities diminished by 10^4^ and 10^3^ times, respectively. Mutants of hMGL, formed by nonconservative replacement of catalytic acid Asp-239 to alanine or asparagine exhibit activities reduced by 137.2 and 87.4 times, respectively.

**Table 1:** Steady-state kinetic parameters for sol-hMGL and active site mutants based on the hydrolysis of endogenous substrate 2-AG.

Protein	*K*_m_ (μmol/L)	*V*_max_ (μmol/(L s)	*K*_cat_ (s^−1^)	Catalytic efficiency (*K*_cat_/K_m_)	Fold decrease^a^	Conformational status at 310 K^b^
sol-hMGL	19.3 ± 2.4	0.55 ± 0.02	5.69 ± 0.16	0.30		Open
S122A	–	–	–	–	–	Open
S122C	97.9 ± 28	(1.57 ± 0.18) × 10^–4^	(1.61 ± 0.17) × 10^–3^	1.64 × 10^–5^	1.8 × 10^4^	Open
S122T	80.7 ± 5	(1.85 ± 0.04) × 10^–3^	(1.89 ± 0.04) × 10^–2^	2.34 × 10^–4^	1.3 × 10^3^	Closed
H269A	–	–	–	–	–	Open $\rightleftarrows$ closed
D239A	95.7 ± 8.1	(2.02 ± 0.07) × 10^–2^	0.21 ± 0.01	2.15 × 10^–3^	137.2	Closed
D239N	42.5 ± 5.7	(1.40 ± 0.05) × 10^–2^	0.14 ± 0.01	3.38 × 10^–3^	87.4	Open
L241A	11.5 ± 2.2	0.19 ± 0.01	1.92 ± 0.08	0.17	1.8	Open $\rightleftarrows$ closed
C242A	69.0 ± 5.5	0.36 ± 0.01	3.71 ± 0.09	5.37 × 10^–2^	5.5	Open $\rightleftarrows$ closed
H121A	23.9 ± 4.5	(6.74 ± 0.33) × 10^–2^	0.69 ± 0.03	2.88 × 10^–2^	10.3	Open $\rightleftarrows$ closed
Y268A	17.9 ± 3.2	0.40 ± 0.02	4.07 ± 0.17	0.23	1.3	Open $\rightleftarrows$ closed

Uncertainties in data are presented as mean ± standard error (1σ). Conformations observed by ^1^H NMR experiments at T = 310 K are listed.

^a^$$Fold\;decrease = {{\left( {{\raise0.7ex\hbox{${K_{{{\text{cat}}}} }$} \!\mathord{\left/ {\vphantom {{K_{{{\text{cat}}}} } {K_{{\text{m}}} }}}\right.\kern-\nulldelimiterspace} \!\lower0.7ex\hbox{${K_{{\text{m}}} }$}}} \right)^{{{\text{sol - hMGL}}}} } \mathord{\left/ {\vphantom {{\left( {{\raise0.7ex\hbox{${K_{{{\text{cat}}}} }$} \!\mathord{\left/ {\vphantom {{K_{{{\text{cat}}}} } {K_{{\text{m}}} }}}\right.\kern-\nulldelimiterspace} \!\lower0.7ex\hbox{${K_{{\text{m}}} }$}}} \right)^{{{\text{sol-hMGL}}}} } {\left( {{\raise0.7ex\hbox{${K_{{cat}} }$} \!\mathord{\left/ {\vphantom {{K_{{cat}} } {K_{m} }}}\right.\kern-\nulldelimiterspace} \!\lower0.7ex\hbox{${K_{m} }$}}} \right)^{{{\text{mutant}}\;{\text{hMGL}}}} }}} \right. \kern-\nulldelimiterspace} {\left( {{\raise0.7ex\hbox{${K_{{cat}} }$} \!\mathord{\left/ {\vphantom {{K_{{cat}} } {K_{m} }}}\right.\kern-\nulldelimiterspace} \!\lower0.7ex\hbox{${K_{m} }$}}} \right)^{{{\text{mutant}}\;{\text{hMGL}}}} }}$$

^b^Conformational status of the gate in hMGL mutants. See text.

Conservative L241A and nonconservative C242A replacements of residues acting as amide hydrogen-bond donors to proximal side chain acceptor Asp-239, resulted in a decrease of catalytic efficiency by 1.8 and 5.5 times, respectively, compared to sol-hMGL. Nonconservative substitutions of the aromatic His-121 residue, preceding the nucleophile, and Tyr-268 residue, preceding the catalytic base for alanine, resulted in the reduction of catalytic efficiency by 10.3 and 1.3 times, respectively.

### Effect of active site residue mutations on NMR spectra and conformational gating of hMGL

Conformational gating is the reversible switching between open and closed conformations, which controls the access of small molecules into the binding pocket of enzyme^38^. As a structural and dynamic system, the gate may synchronize processes occurring at the active and distal sites of enzyme. The gating event could be affected by site-directed mutagenesis via shifts in the populations of conformational states. NMR spectroscopy can directly detect such population changes.

Previously we established using 1D ^1^H NMR spectroscopy that hMGL displays five distinct and well separated downfield resonances (11–16 ppm)^35^, four of which were unambiguously assigned to the His-103 (15.9 ppm), His-269 (14.9 ppm), His-54 (13.9 ppm) and His-49 (12.8 ppm). The resonance at 11.5 ppm belongs to an unassigned OH group. These downfield resonances represent a ^1^H NMR spectral pattern of the open (active) hMGL form. These residues are highly sensitive to the open-closed interconversions of the enzyme and to ligand binding events. During the open-to-closed transition, His-54 and His-269 peaks experience significant upfield shifts that are well outside of this downfield region. As a result, the spectral pattern for the closed form differs significantly from the open form by absence of these two peaks^35^. Thus, under slow exchange conditions on the NMR time scale, distinct NMR patterns allow unambiguous identification and quantification of the open and closed conformers in mixtures upon changes of physical environment, mutations and ligand binding. Table 1 summarizes the observed relative population of active and inactive conformations of each mutant at physiological temperature 310 K.

**Figure 2:**
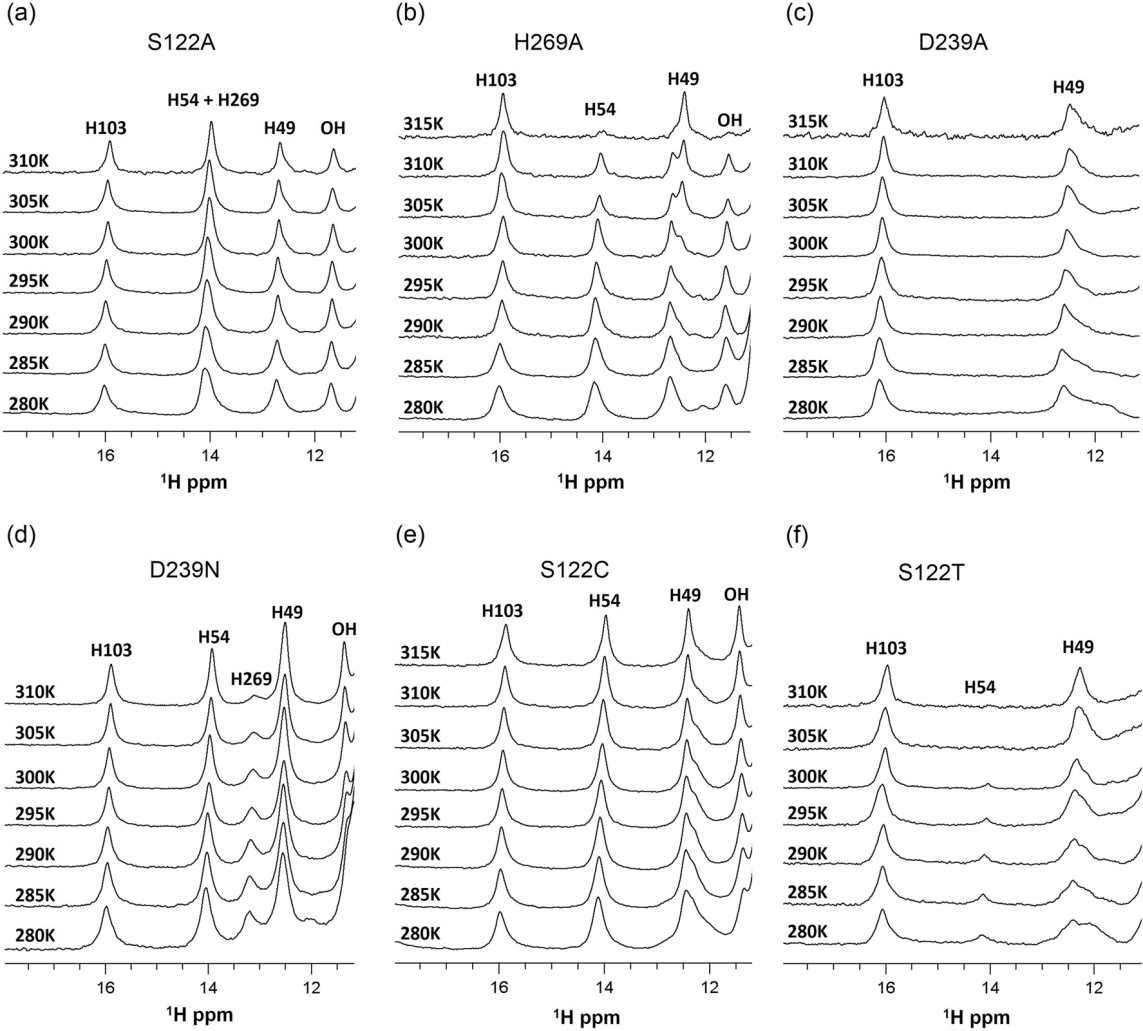
Downfield ^1^H NMR spectra of catalytic triad mutants demonstrating effects of single-point mutations and temperature on the relative population of open and closed conformers of hMGL. (**a**) S122A, (**b**) H269A, (**c**) D239A, (**d**) D239N, (**e**) S122C and (**f**) S122T.

We explored the effects of nonconservative residue substitutions in the catalytic triad on conformational gating. The downfield region of NMR spectrum for the S122A construct (Fig. 2a) shows distinct resonances that resemble the pattern of the open conformer except that the His-269 resonance peak, due to its upfield shift, has merged with His-54 resonance peak. This NMR spectrum suggests minor conformational rearrangement within the catalytic triad, and this local perturbation does not induce global conformational changes associated with detectable population shift. Temperature variation within the range 280 K to 310 K did not induce any conformational shift, thus, demonstrating the stability of the open conformations in the S122A mutant. Therefore, the hMGL gating mechanism can tolerate nonconservative mutation at position 122, suggesting that the surrounding networks of interactions can compensate for nucleophile substitution. Although this populates open conformations, S122A exhibits no catalytic activity. The absence of a nucleophile in the catalytic triad accounts for the inactivity of this hMGL construct favoring open forms.

In contrast to the S122A mutation, substitution of catalytic base His-269 by alanine triggers the modulation of conformational gating, resulting in a substantial population shift toward closed states. The downfield region of spectra in this case consists of two NMR patterns corresponding to open (major) and closed (minor) conformations. As the temperature is elevated from 280 to 310 K, ^1^H NMR spectra of the catalytically inactive H269A construct (Fig. 2b) exhibit diminishing His-54 resonances, indicating a gradual decrease in the population of open forms. However, lowering the temperature favors the open conformers. All spectral changes are reversible with respect to temperature. The observed destabilization of open (active) conformers at physiological temperature may be attributed, in part, to the breaking of the catalytically important Asp-239–His-269 hydrogen bond as a result of catalytic base removal.

Notably, the D239A construct, formed by the nonconservative substitution of aspartic acid by alanine in the catalytic triad, exhibits a ^1^H NMR pattern indicative of closed conformers (Fig. 2c). The observed stabilization of closed forms is due to the simultaneous disruption of the three hydrogen bonds to the substituted aspartate side chain, including the critical Asp-239 and His-269 hydrogen bond. The ^1^H NMR spectra observed between 280 and 310 K exhibit no evidence of equilibrium reversibility, suggesting substantial modulation of conformational gating upon disruption of a preorganized hydrogen bonding network. Thus, nonconservative substitution of the catalytic acid by alanine induces the major conformational shift toward closed forms. This result highlights the important role that Asp-239 plays in conformational gating of hMGL.

D239N, formed by substitution of catalytic aspartic acid by asparagine, alters the downfield ^1^H NMR pattern by an upfield shift of the H269 resonance from 14.9 to 13.1 ppm (Fig. 2d). This suggests formation of an alternative hydrogen bond between Asn-239 and His-269 instead of the original Asp-239–His-269 hydrogen bond, despite expected geometry modification at the active site. The ^1^H NMR spectra of D239N mutant observed between 280 and 310 K provide clear evidence that the open conformers are stable over a wide range of temperature. The conformational stability and catalytic activity of the D239N indicates that the asparagine residue can, to some extent, replace Asp-239 and maintain proper conformational gating of hMGL.

The impact of conservative substitutions of the catalytic triad residues on the molecular mechanism of gate function was explored. Substitution of nucleophile Ser-122 to cysteine formed S122C containing a catalytic triad similar to those found within cysteine proteases^35^. In this case, the cysteine nucleophile donates a proton to the catalytic base His-269, forming an imidazole-thiolate ion pair between the His-269 and Cys-122 in the modified active site of hMGL. The NMR spectra showed a significant downfield shift of the His-269 signal from 14.9 to 18 ppm, indicating that the catalytic histidine is in a protonated state (Fig. S5). Temperature dependent NMR spectra of S122C, observed between 280 and 315 K (Fig. 2e), show the stability of the open conformations and provide evidence that hMGL conformational gating is not affected by this conservative replacement.

The S122T mutant is formed by the conservative replacement of the serine nucleophile to threonine. At 310 K, S122T NMR spectrum indicates a population of closed conformers only (Fig. 2f). As the temperature decreases to 280 K, the emergence of the His-54 peak in the NMR spectra points to the presence of detectable amounts of open conformers, indicating the reversibility of closed-open equilibrium. This result demonstrates that conservative substitution of the nucleophile with threonine was not compensated by the catalytic triad surroundings, causing substantial perturbations in the mechanism of conformational gating. The data from these hMGL mutants clearly highlight the presence of a delicate dynamic equilibrium between the open and closed conformers of hMGL, which is highly sensitive to the stereochemistry and chemical properties of the substituting residue.

**Figure 3:**
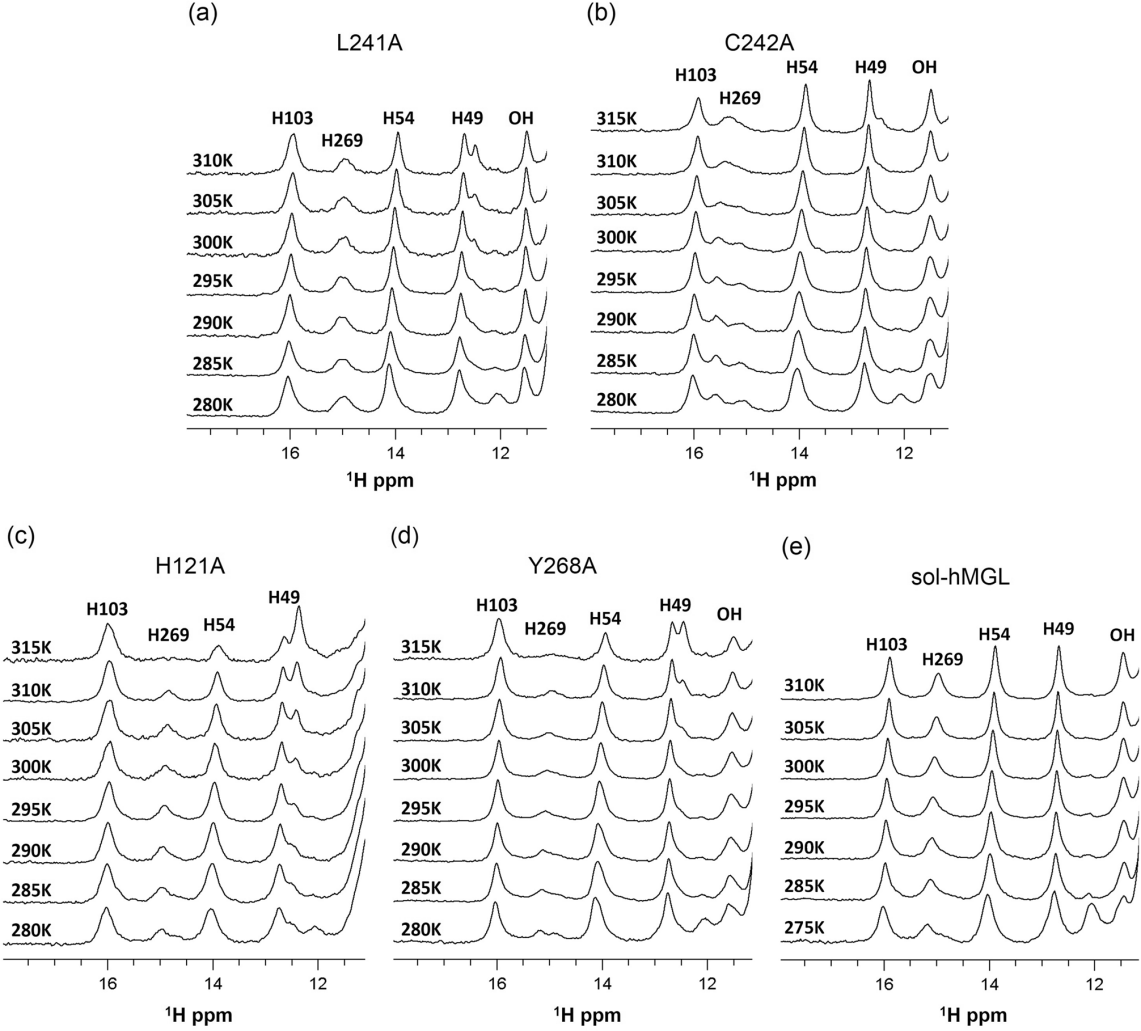
Downfield ^1^H NMR spectra of L241A and C242A mutants with modulated hydrogen bonding to Asp-239, and H121A and Y268A mutants with affected aromatic interactions, demonstrating effects of single-point mutations and temperature on the relative populations of open and closed conformers of hMGL. (**a**) L241A, (**b**) C242A, (**c)** H121A, (**d**) Y268A and (**e**) sol-hMGL.

We further investigated the role of two backbone amide groups of Leu-241 and Cys-242, both of which act as hydrogen bond donors to Asp-239 O^δ1^. These amides may serve as anchor groups for the stabilization and proper orientation of the catalytic acid (Fig. 1), and thus, may participate in the active site “fine-tuning” rearrangements during catalysis. The NMR spectra for the L241A (Fig. 3a) and C242A (Fig. 3b) mutants clearly show the presence of conformational equilibria between the open and closed forms. For both constructs increased temperature shifts the populations partially toward the closed forms. At 310 K the ^1^H NMR spectral patterns are consistent with a major population of the open forms. The substitutions at positions 241 and 242 led to a 1.8- to 5.5-fold decrease in catalytic efficiency, respectively, indicating involvement of Leu-241 and Cys-242 in the regulation of hMGL conformational gating.

Mutants, H121A and Y268A, allow for the explorations of the potential influence of aromatic interactions in stabilizing the catalytic triad and regulation of conformational gating. NMR spectra of these mutants (Fig. 3c,d) at 310 K display the downfield resonances consistent with a major population of the open conformers and a detectable amount of the closed forms. Temperature dependent experiments demonstrate the existence of reversible equilibria between the open and closed conformers. Similar to H269A, L241A, and C242A substitutions, decreasing the temperature stabilizes the open (active) conformers whereas increasing the temperature stabilizes closed (inactive) conformers of enzyme. These results indicate that aromatic interactions contribute to the preorganization of the catalytic triad and mediation of conformational gating. The temperature dependent ^1^H NMR spectra (downfield regions) of sol-hMGL (Fig. 3e) demonstrate stability of open conformers for this construct in a wide range of temperatures and allow comparative analysis of results obtained for all mutants (Figs. 2 and 3).

### Accessibility of ligands to the active site of hMGL mutants

Dynamic modulation of hMGL conformational gating by point mutations could potentially slow down ligand binding, as it may directly affect the accessibility of ligands into the active site^39^. Earlier, we have shown that chemical shift perturbations (CSPs) in the downfield region of ^1^H NMR spectra are indicative of ligand specific binding and consequent conformational rearrangements^37^. Thus, CSPs upon binding to a known high affinity ligand will provide straightforward information on ligand accessibility to the active site of hMGL. In turn, this information will be useful for understanding the role of specific inter-residue interactions in conformational gating of the enzyme.

**Figure 4:**
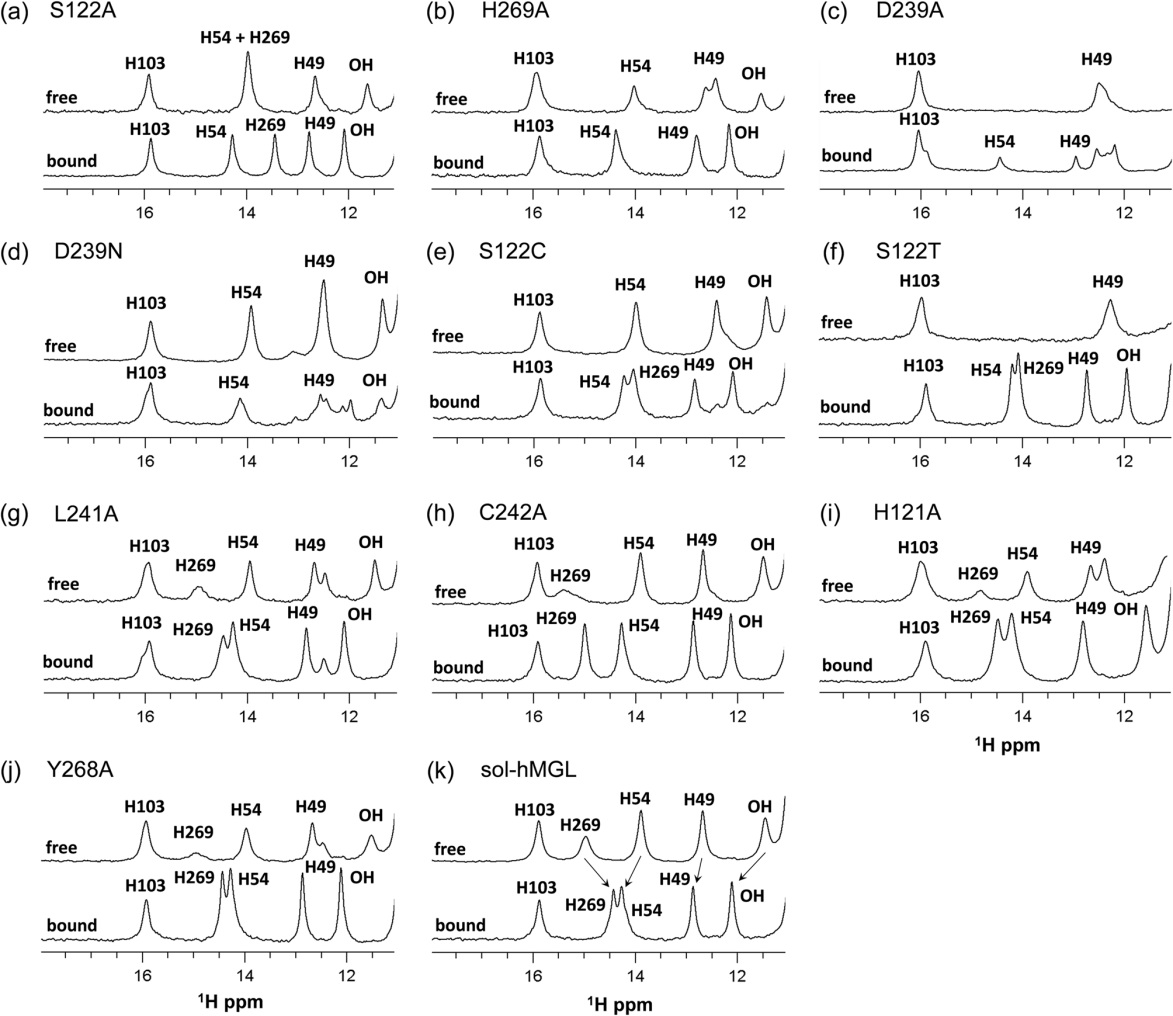
The downfield ^1^H NMR spectra of mutants and sol-hMGL in free and bound with compound 1 forms, providing evidence for ligand accessibility to the active site and demonstrating the relative degree of binding site occupancy. (**a**) S122A, (**b**) H269A, (**c**) D239A, (**d**) D239N, (**e**) S122C, (**f**) S122T, (**g**) L241A, (**h**) C242A, (**i**) H121A, (**j**) Y268A and (**k**) sol-hMGL.

As in previous work^37^, we used compound 1 (2-cyclohexyl-6-{[3-(4-pyrimidin-2-ylpiperazin-1-yl)azetidin-1-yl]carbonyl}-1,3-benzoxazole) as a potent and reversible (K_i_ ~ 10 nM) hMGL inhibitor^3^ to probe ligand accessibility into the active site of the enzyme. CSPs in the characteristic NMR pattern of the downfield ^1^H NMR spectrum for sol-hMGL upon formation of reversible complex with compound 1 are shown in Fig. 4k. Similar changes for His-269, His-54, His-49 and OH resonances were observed for all mutants, indicating ligand penetration into the active site as well as the degree of binding site occupancy (Fig. 4a–j). For S122A, H269A, S122C, H121A, and Y268A substitutions, complete complex formation was observed at an enzyme/ligand mole ratio of 1:1; a twofold excess of compound 1 for C242A and fourfold excess for S122T and L242A are needed to achieve a complete complex formation. In the case of D239A and D239N substitutions, even a significant excess of the ligand (1:10 mole ratio) did not shift the equilibria completely towards complex formation (Fig. 4c,d). This fact suggests substantial structural and dynamic modulation of enzyme through substitutions at position 239, which highlights the essential role of Asp-239 in the mechanism of hMGL conformational gating.

### Global conformational and dynamic changes upon ligand binding

2D ^1^H–^15^N HSQC NMR binding data (Fig. S3 and details in SI) may be explained on the basis of a conformational selection model. Upon addition of ligand to the equilibrium mixture of conformers, the ligand binds only to the open (active) forms shifting the preexisting equilibrium to the open forms. Substantial occupancy of active sites for D239A, observed in the presence of an excess of ligand, provides evidence for the conformational shift toward the open forms and robustness of the hMGL active site. The identified robustness suggests the existence of supporting structural scaffolds that coordinate the catalytic triad residues and may compensate for significant modulation of conformational gating. The minimal compensation revealed for D239A substitution highlights the essential role of the catalytic acid in the mechanism of hMGL conformational gating.

### HDX-MS analyses of hMGL dynamics upon substitution of catalytic triad residues

To directly investigate the dynamics of different regions of hMGL upon mutations, we have used the HDX-MS methodology that has been well established^40,41^. At the peptide length resolution these data provide insight into the changes of hMGL dynamics induced by mutations.

**Figure 5:**
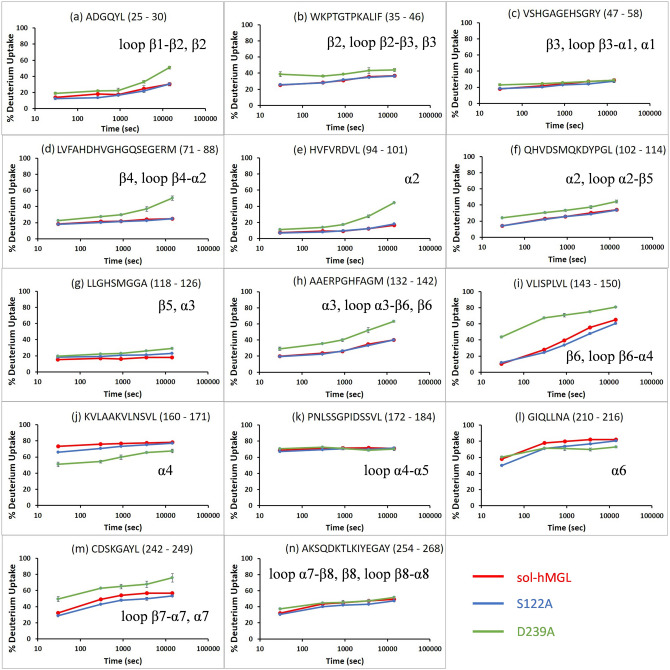
Plots of deuterium uptake vs log(*t*) (30 s, 5 min, 15 min, 1 h, 4 h) for the 14 peptides (**a**–**n**) at 298 K, showing statistically different behavior for D239A mutant as compared to sol-hMGL and S122A mutant. The annotation on each panel indicates the amino acid sequence and the structural feature (ref. Fig. 1) associated with the peptide. For time points with uncertainties larger than the plotted symbol, bars indicate the measurement uncertainty (1σ). Peptic peptides: (**a**) 25–30, (**b**) 35–46, (**c**) 47–58, (**d**) 71–88, (**e**) 94–101, (**f**) 102–114, (**g**) 118–126, (**h**) 132–142, (**i**) 143–150, (**j**) 160–171, (**k**) 172–184, (**l**) 210–216, (**m**) 242–249 and (**n**) 254–268.

Peptic peptides for sol-hMGL, S122A, and D239A were identified from MS/MS spectra, and the peptides are plotted as bars against the respective protein sequences in Figs. S6–S8. HDX-MS data for these species share a set of 53 common peptides, which covers 80% of the hMGL sequence with some redundancy. To remove redundancy, the larger set was reduced to a subset of 23 peptides that fulfills maximum sequence. The reduced subset allows for head-to-head comparison of deuterium uptake vs time plots for peptides of S122A, D239A, and sol-hMGL (Fig. 5). Table S1 lists the deuterium uptake (D-uptake) for the peptides as a function of time. The listed uncertainties denote the standard deviations (1σ), which are also graphically shown by bars in Fig. 5. Within the resolution of the peptides the D-uptake data reveal that the dynamics of S122A and sol-hMGL are approximately identical and that D239A is more dynamic in a larger number of regions along the protein sequence.

We first analyze peptides from the catalytic triad zone. Specifically, peptide 118–126 (Fig. 5g), containing the nucleophile Ser-122 and the oxyanion hole residue Met-123, demonstrates small rates of deuterium exchange for sol-hMGL and both mutants, indicating that this region is conformationally stable and highly protected from exchange. This result is consistent with the known conformational rigidity of the nucleophilic elbows within serine proteases (sharp turn between strand β5 and helix α3 for hMGL) where the catalytic serine resides. Peptide 118–126 from the D239A mutant exhibits slightly more dynamic D-uptake vs log(*t*) behavior at all D_2_O incubation, indicating that the mutated catalytic triad is less protected from H/D exchange. However, the relatively small amount of dynamic perturbation indicates a structure that is maintaining conformational stability, proving the importance of rigidity and dynamic stability at the hMGL active site. Similarly, partially protected peptide 254–268 (Fig. 5n), which includes the loop (266–268) preceding the catalytic base His-269, shows only slight changes in D-uptake in both mutants. For both mutants only slight changes are observed in D-uptake data for the functionally important peptide 47–58 (Fig. 5c). This peptide reports D-uptake in the parts of strand β3 and helix α1, and in the loop connecting β3 with α1, where the structurally conserved, oxyanion hole-forming residues, Gly-50 and Ala-51, reside. The small variations in D-uptake by residues that report on the catalytic triad provide additional support for the concept of active site dynamics stability.

Peptide 242–249 (Fig. 5m), containing the loop between strand β7 and helix α7, following the catalytic acid Asp-239, exhibits a substantial stepwise increase in D-uptake (19%) for the D239A mutant. The increased dynamics in D239A is likely a consequence of breaking of the critical Asp-239–His-269 hydrogen bond in the catalytic triad, resulting in significant alterations in the conserved hydrogen-bonded network of the active site. In the S122A mutant, peptide 242–249 exhibits a slightly reduced D-uptake rate. Taken together, these results suggest that distinctive structural motifs coordinating the catalytic nucleophile with oxyanion hole^34^ and catalytic acid residue^33^ can compensate for structural perturbations caused by direct mutations of catalytic Ser-122 and Asp-239 in the regions of nucleophile, oxyanion hole and catalytic base. Therefore, as evidenced in the dynamics, the existing preorganization of these functionally important regions is not compromised substantially.

Turning to the dynamics of the lid domain, rolling motions of helix α4 over the entrance to the active site of hMGL are proposed to facilitate the open-closed transitions in hMGL^3^. Peptide 160–171 (Fig. 5j) reports on the H/D exchange dynamics of helix α4 in the lid domain. Sol-hMGL and S122A mutant, which are in open conformations, exhibit more rapid D-uptake rates than observed for D239A in the closed conformation. The closed conformation of D239A is stabilized by the hydrophobic side of helix α4 turning toward the catalytic pocket, resulting in partial burial. The burial into the solvent-shielded hydrophobic interior of hMGL stabilizes D239A against H/D exchange. The suppressed D-uptake rate of peptide 210–216 (Fig. 5l) suggests that the burial involves helix α6.

Lid domain helix α4 links via a loop and strand β6 to the α/β-hydrolase core domain of hMGL. Peptide 143–150 (Fig. 5i) reports the D-uptake vs log(*t*) of this loop. The D-uptake traces of sol-hMGL and S122A mutant are similar. Both hMGL constructs exhibit a nearly linear increase from 10% D at *t* = 30 s to 60% D at *t* = 4 h, indicating that the associated residues are moderately protected against H/D exchange. In contrast, the D239A mutant exhibits a bimodal D-uptake function comprising rapid initial D-uptake of 43% D at *t* = 30 s and 70% D at *t* = 5 min, which moderates to a slower linear increase to 80% D at *t* = 4 h. Thus, relative to sol-hMGL and S122A, strand β6 of D239A is more dynamic, which affects the dynamic behavior of helix α4 directly.

Peptide 132–142 (Fig. 5h) reports on the temporal D-uptake of helix α3, which is linked to strand β6. Sol-hMGL and S122A exhibit nearly identical D-uptake vs. log(*t*) traces that increase from 20% D at *t* = 30 s to 40% D at *t* = 4 h, indicating that the associated residues have moderate protection against H/D exchange. In contrast, temporal D-uptake for peptide 132–142 in D239A rises from 20% D at *t* = 30 s to 40% D at *t* = 4 h, indicating that the associated residues are less protected and more dynamic. The increased D-uptake rate observed in strand β6 and helix α3 of D239A relative to sol-hMGL evidences increased dynamics associated with structural changes that affect conformational gating. These changes in dynamics may result in a shift of equilibrium toward closed conformations with restricted access to the binding pocket.

Lid domain helix α4 also links to helix α5 via a long loop. Peptide 172–184 (Fig. 5k) reports the temporal D-uptake along this loop. The D-uptake traces for all hMGL constructs exhibit nearly identical behavior, comprising a jump to 65% D at *t* = 30 s with minimal change subsequently. Thus, D-uptake in this loop is not affected by either mutation. This invariance evidences the highly dynamic nature and high level of solvent exposure in the associated residues. Collectively, the data for peptides derived from structural regions connecting the lid domain with the α/β-hydrolase core of hMGL strongly suggest that D239A substitution induces substantial structural rearrangements.

Closer to the N-terminus of the enzyme, we observed five peptides that display substantially greater the D-uptake for the D239A mutant as compared to the sol-hMGL and S122A constructs. Peptide 25–30 (Fig. 5a) reports on the loop connecting strand β1 with β2 and a portion of the β2 strand. Compared to the same peptide in the sol-hMGL and S122A constructs, peptide 25–30 shows greater amide D-uptake at all exchange times, especially at *t* = 4 h (≈ 30% D in sol-hMGL and S122A vs. 51% D in D239A). Similar behavior was observed for peptide 71–88 (Fig. 5d), which samples strand β4 and part of the loop connecting β4 with helix α2 (25% D in sol-hMGL and S122A vs. 50% D in D239A at *t* = 4 h) as well as for peptide 94–101 (Fig. 5e), which samples part of helix α2 (16% D vs. 44% D at *t* = 4 h). Additionally, peptide 35–46 (Fig. 5b), which samples part of strand β2, the loop connecting β2 with β3, and part of strand β3, and peptide 102–114 (Fig. 5f), which samples part of helix α2 and the loop between α2 and strand β5, also demonstrate greater D-uptake for D239A mutant than for the sol-hMGL and S122A constructs. All together, these peptides from remote, distinct regions of enzyme provide evidence for global changes in conformational dynamics upon substitution of the catalytic acid.

**Figure 6:**
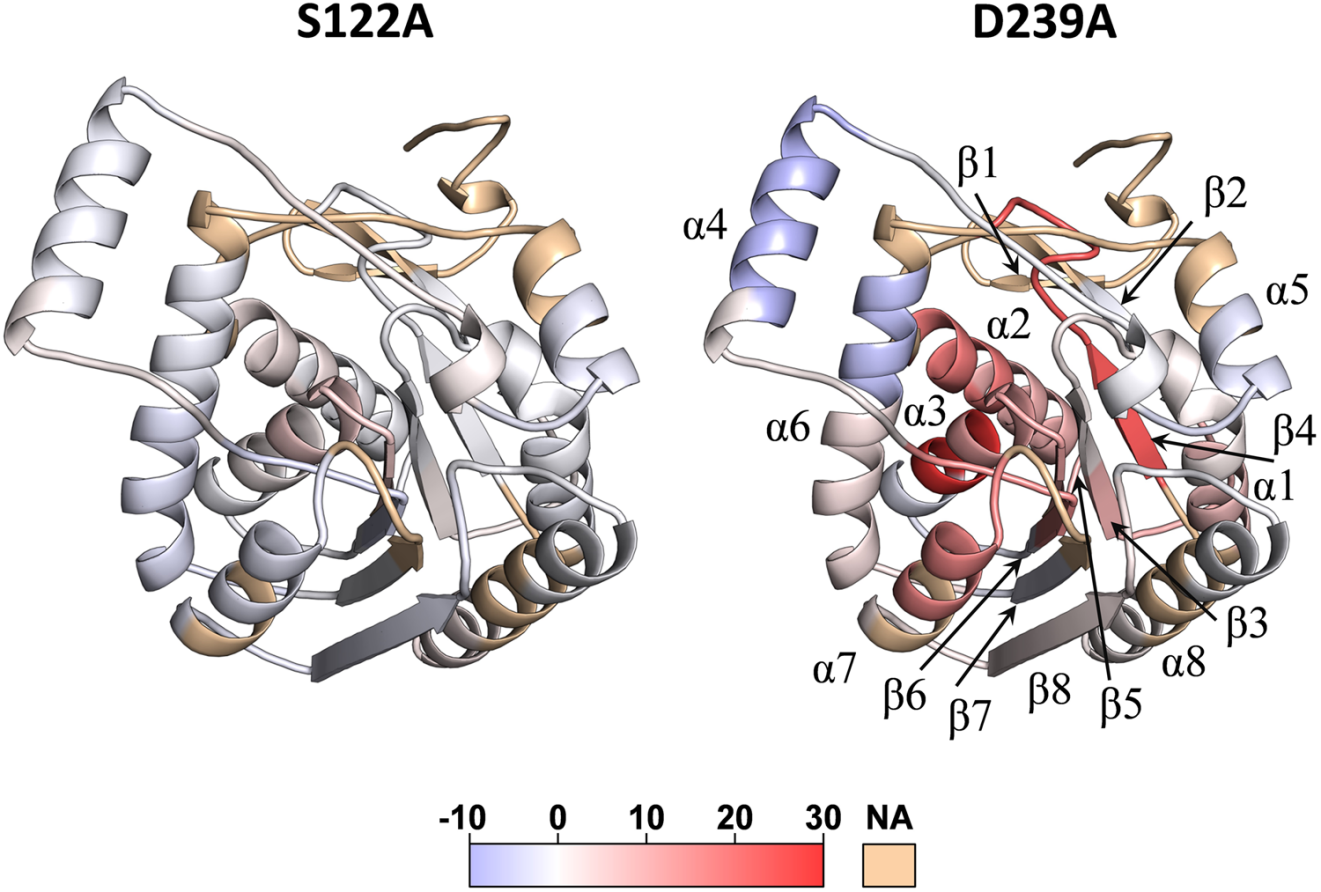
Differential deuterium uptake profiles at *t* = 4 h of S122A and D239A, referenced to sol-hMGL, which are mapped onto the crystal structure of hMGL (PDB: 3HJU). Increased differential deuterium uptakes are color-coded with gradual color change from white to red and reduced uptakes from white to blue. Light yellow indicates regions where peptic peptides were not detected.

Insight into the nature of hMGL conformational changes is obtained by mapping the positions of differential deuterium uptakes onto the structure (Fig. 6). Regional differential deuterium uptake profiles for S122A and D239A mutants are color-coded with gradual color change onto the enzyme’s wild type X-ray crystal structure. The profiles for both mutants were calculated as a difference between the percent amide deuterium content in sol-hMGL and mutant at *t* = 4 h exchange duration.

In summary, HDX-MS data indicate that hMGL maintains structural integrity when the nucleophile or catalytic acid is substituted. Substantial dynamical differences for a notable fraction of hMGL residues from distinct regions of D239A mutant indicate a state of increased dynamic flexibility and major conformational shift toward the closed states.

### Metadynamics simulations and free energy landscapes (FELs) for catalytic triad mutants of hMGL

**Figure 7:**
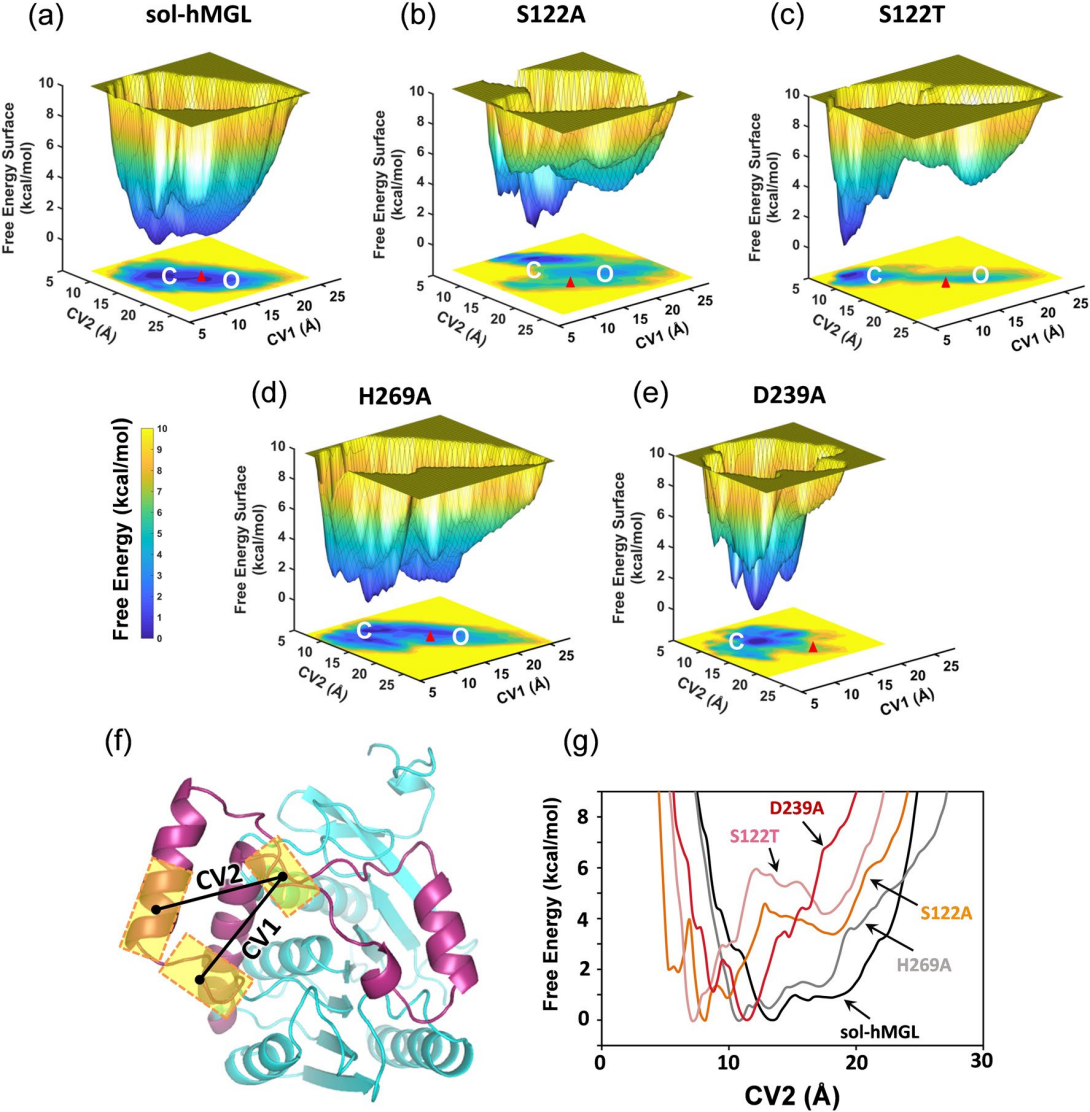
Comparison of the FELs of sol-hMGL and catalytic triad mutants: (**a**) sol-hMGL, (**b**) S122A, (**c**) S122T, (**d**) H269A, and (**e**) D239A. The letter “O” on the *CV1*-*CV2* plane marks the free-energy minimum corresponding to the enzyme with the open lid domain, and the letter “C” marks the free-energy minimum of enzyme with closed lid domain. A red triangle marks the values of *CV*s determined from the X-ray structure of hMGL in open conformation (PDB 3HJU). The vertical color bar represents the scale of free energy values in the unit of kcal/mol. (**f**) The lid domain portion of enzyme is highlighted in magenta, and residues selected for localization the centers of mass of C_α_ carbons are represented by yellow rectangles. The *CV*s distances are shown by black solid lines. Centers of mass are marked by black dots. (**g**) Free energy (ΔG) vs. *CV*2 for each mutant.

To gain additional insights into the gating mechanism of hMGL, metadynamics simulations were performed. We evaluated the effects of catalytic triad residue mutations on the conformational free energy landscape (Fig. 7) using well-tempered (WT) metadynamics with the OPLS3e force field^42^. It is well recognized that FELs can be used to explain protein function, catalysis and allostery^43–47^. Based on the two X-ray crystal structures of hMGL with open^29^ and with closed lid domain^3^, we have selected the variables *CV1* and *CV2*, which represent distances between the centers of mass of C_α_ carbons in residues forming lid domain rims. Specifically, *CV1* is the distance between the centers of mass of C_α_ carbons of residues 176–179 and 150–157, whereas *CV2* represents the distance between residues 176–179 and 158–164 (Fig. 7f). These collective variables allow us to discriminate efficiently between two extreme conformations of hMGL^8^. The conformational transition from open to closed state will reduce the distances between the lid domain rims, which could be indicated by lowering of *CV* values on the FEL diagrams. Metadynamics exploration revealed that hMGL can undergo dynamic equilibrium between different stable conformations residing within the free-energy wells. During each of the 350 ns simulations, we observed interconversion between open and closed conformations for sol-hMGL, S122A, H269A and S122T, but not D239A as this mutant primarily adopted closed conformations.

The FEL diagrams show that the catalytic triad residue mutations induce population shifts to different extents, depending on positions as well as types of residue. Though the FELs for all constructs (Fig. 7a–e) exhibit rugged and complex surfaces, two major free-energy wells are identified in the global free-energy regions of the analyzed constructs, except for the D239A mutant, which shows only one major well. These results suggest that substitutions in the catalytic triad of hMGL may alter relative populations of the different conformations or even shift the equilibrium toward one extreme conformation. For the sol-hMGL construct, the free energy difference between the open (O) and closed (C) states is rather insignificant (0.9 kcal/mol, Fig. 7a). For the nonconservative S122A and conservative S122T substitutions at the nucleophile residue position, substantial shifts of conformational equilibria are observed due to the stabilization of hMGL in the closed conformation (Fig. 7b,c). The energy difference between these two conformations increases significantly, 3.4 kcal/mol for S122A and 4.2 kcal/mol for S122T, respectively.

Interestingly, when referenced to sol-hMGL, the free energy minimum of the open form for both S122A and S122T mutants have larger *CV1* and *CV2* distances, corresponding to conformations with more-open lids (super-open). For the catalytic base residue mutation H269A the nonconservative substitution induces a more rugged surface than observed for sol-hMGL without notable alteration of conformational distribution (Fig. 7d). However, a similar substitution of catalytic acid residue Asp-239 for alanine shifts the equilibrium completely towards the closed conformations (Fig. 7e), which may be recognized as a typical result of allosteric mutations.

## Discussion

Our observation of the redistribution of conformational equilibria in hMGL, triggered by environmental perturbations (e.g. temperature variation, pH change), ligand binding, and mutations, provides a more complete understanding of the allosteric regulation of the enzyme’s function^35,37^. Moreover, identification of critical interactions and residues essential for allosteric communications may facilitate the discovery of allosteric inhibitors targeting the conformational transitions, population shifts, or flexibility of hMGL. Here, we have used a comprehensive set of experimental approaches and metadynamics simulations to gain insight into the mechanisms of residue-specific interactions, conformational gating, and allosteric communications of hMGL. The recognition that the equilibrium structure is an ensemble of conformations, but not a single structure, allows us to describe enzymes in terms of the shift in conformational population and in terms of free energy landscape at a more fundamental level^48^. The allosteric communication is not obvious through simple comparisons of the open and closed crystal structures. Instead, it can be defined by exploring the basic principles of allostery, including pre-existence of conformational states and changes in the conformational landscape as a function of different effectors. Therefore, understanding the conformational dynamics and allosteric couplings between residues is essential to elucidate the molecular basis of physiological regulatory mechanisms and allosteric inhibition of hMGL.

In this work, we used highly sensitive downfield ^1^H NMR resonances, which represent the best atomic resolution probes, to sample population distributions of active and inactive conformers of hMGL to reveal subtle allosteric perturbations. In addition to NMR spectroscopy, HDX-MS was used as an effective technology for the identification of distinct regions that may mediate intraprotein allosteric communications. Due to their critical role in catalysis, the catalytic triad and its proximal residues constitute proper targets for mutagenesis experiments to reveal the structural and dynamic rearrangements and to elucidate the role of regulatory residues responsible for modulation of hMGL activity.

We have found that while showing drastic decreases in the catalytic efficiency, nonconservative substitutions of catalytic triad residues demonstrate highly varied impacts on the conformational equilibria of hMGL. The S122A mutation does not induce a detectable population shift of conformational equilibrium. In the case of H269A substitution, the population is partially shifted toward the closed conformers. Likewise, D239A substitution demonstrates a complete shift toward the closed conformations. In contrast, D239N substitution does not affect equilibrium, demonstrating stability of open conformers over a wide range of temperatures. Interestingly, conservative substitutions in the catalytic triad also produce different effects on the conformational balance. While S122C mutation does not affect conformational equilibrium, S122T mutation almost completely shifts the equilibrium toward closed conformations.

As to the catalytic triad proximal residues, substitutions of hydrogen bond donors (Leu-241, Cys-242) and aromatic residues (His-121, Tyr-268) to alanine also demonstrate different extent of conformational shifts toward the closed conformations, depending on the nature of residue-specific interactions. None of these mutations caused a dramatic loss of function. These mutants exhibited only minor decreases in catalytic efficiency, which is consistent with subtle population shifts. Taken together, our findings provide experimental evidence that a preorganized network of hydrogen bonds and aromatic interactions within the catalytic triad controls the conformational equilibria between active and inactive hMGL states. This network also accounts for the robustness of the hMGL active site towards point mutations.

To further verify the reversibility of conformational shifts induced by point mutations, NMR ligand binding experiments were performed. Our data provided clear evidence for ligand accessibility to the binding pocket of all hMGL mutants, advocating reversibility of their conformational shifts. Notably, partial reversibility of equilibrium was found only for D239A mutation, suggesting significant modulation of conformational gating, which underlines the critical role of the catalytic acid in the regulation of hMGL activity. Moreover, 2D ^1^H–^15^N HSQC NMR experiments for catalytic triad mutants revealed that ligand binding to the active site caused chemical shift perturbations for a large fraction of the enzymes’ resonances, highlighting the robustness of hMGL conformational gating.

With the aim to identify the role of aromatic amino acids around the catalytic triad, the pairs of residues proximal in space were examined for potential involvement in the allosteric regulation of the enzyme’s function. Nonconservative substitution Y268A caused only subtle effects on conformational equilibrium and catalytic efficiency. More pronounced effects on the same parameters were observed for both H121A and H269A substitutions. These three aromatic residues, His-121, His-269, and Tyr-268, likely contribute to the conformational gating as fine tune regulators during switching between active and inactive hMGL states.

**Figure 8:**
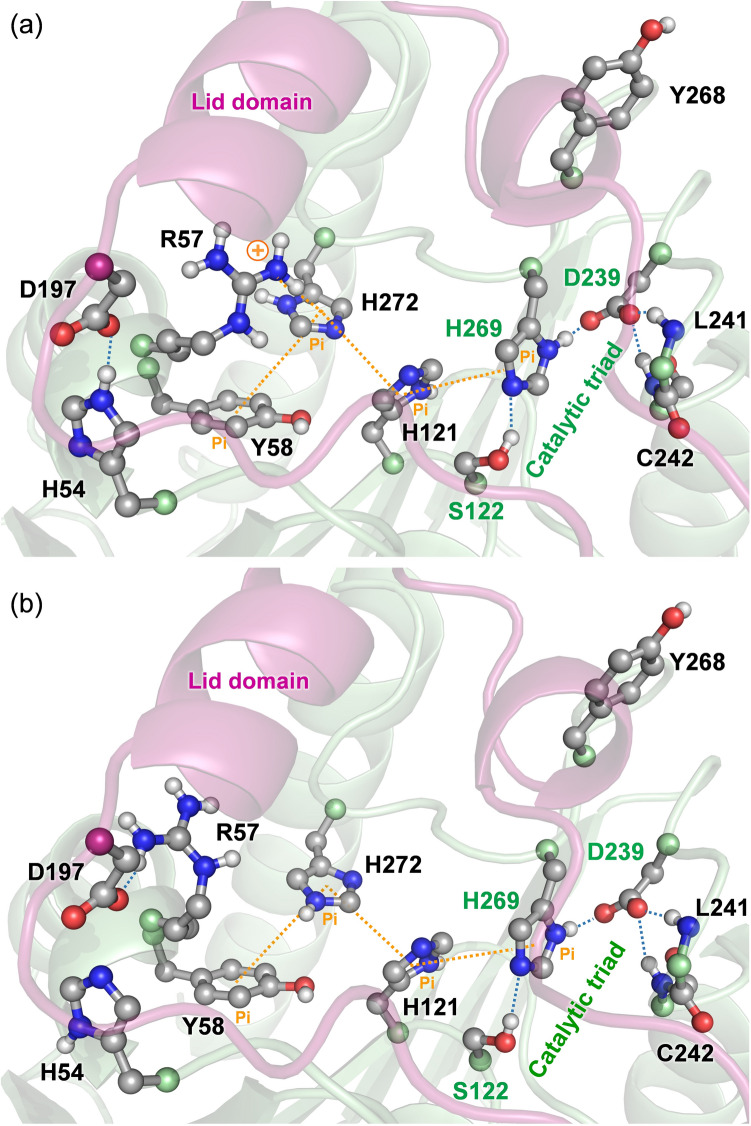
The tertiary network of residue-specific interactions in the active site of hMGL including hydrogen bonding and different type of aromatic interactions. (**a**) cation-π interaction between Arg-57 (arginine switch) and His-272, and hydrogen bond between Asp-197 and His-54 stabilize the open conformations (PDB 3HJU), (**b**) closed conformations are stabilized when Arg-57 flipped away from His-272 and the hydrogen bond between Asp-197 and His-54 is broken (PDB 3PE6). Rearrangements of aromatic interactions are synchronized with hydrogen bonding rearrangements.

Earlier, we also identified His-272 and Tyr-58 as aromatic residues critical for regulation of hMGL activity. Nonconservative substitution H272A caused substantial modulation of hMGL conformational gating^35^. Taken together, our results provide evidence that residues His-121, His-269 and Tyr-268, as well as His-272 and Tyr-58 with Arg-57 as a switch, comprised of an extended aromatic cluster surrounding the catalytic triad. These residue-specific couplings offer mechanistic insights into potential allosteric regulation within the active site of hMGL. Specifically, His-272 is involved in a parallel displaced π-π stacking interaction with Tyr-58 and could also be involved in the edge to face π-π interaction with His-121. Simultaneously, His-272 participates in the cation-π interaction with the cationic –NH_2_^+^ group of Arg-57. This configuration provides effective stabilization of open conformers (Fig. 8a).

When the Arg-57 guanidinium group is flipped away from His-272 toward the Asp-197, the cation-π interaction is disrupted and a new hydrogen bond between NH1 of Arg-57 and O^δ2^ of Asp-197 is formed. As a consequence, the imidazole ring of His-272 may flip 180°, which can introduce conformational rearrangements of His-121 and further propagate via aromatic interactions toward the catalytic base His-269 (Fig. 8b). The above functional couplings identified between residues via different aromatic interactions are synchronized with proximal hydrogen bonding rearrangements and result in the formation of the tertiary communication network within the active site of enzyme. This highly preorganized network of tertiary contacts, including both the hydrogen bonding network and the aromatic interactions, may facilitate functional coupling between structural rigidity and conformational mobility of the catalytic triad. These interactions are likely responsible for the optimal dynamics in the enzyme, which is critical for activation of hMGL.

Molecular details underlining the intraprotein allosteric communications in hMGL were revealed from the alterations in dynamics of different regions. Our HDX-MS data showed that the enzyme maintains structural integrity when the nucleophile or catalytic acid was substituted for alanine. However, substantial differences in dynamics were found for a notable fraction of hMGL residues in the D239A mutant, indicating a state of increased dynamic flexibility and a major conformational shift toward the closed states. Remarkably, peptides 132–142, and 143–150, derived from the structural region connecting the lid domain with the hMGL core demonstrate the largest increase in deuterium incorporation, suggesting major structural rearrangements in this area. Furthermore, many peptides residing in the remote regions, including the N-terminal, also demonstrate substantial increases in deuterium uptake, providing evidence for global structural and dynamical alterations induced by substitution of catalytic acid. These contiguous regions with increased dynamics include not only loops but also regular secondary-structure elements. Therefore, it is reasonable to conclude that rigid α-helixes and β-strands and the connecting flexible loops are involved in communication networks between the active site and various remote regions, serving as channels for long-range signal transmission. Thus, conformational changes within the catalytic triad of hMGL can be transmitted to the distal regions via structural and dynamical alterations in the flexible and rigid elements of entire enzyme. This finding implies the existence of a global communication network, combining both tertiary (inter-residue contacts) and quaternary (rigid-body contacts) communication networks.

It was hypothesized earlier that allosteric communication in proteins can occur via integrated networks of tertiary and quaternary motions^49^. Our data provide experimental support for this hypothesis. Considering the existence of a preorganized global communication network, the substantial increase in dynamics of many regions in D239A mutant and the consequent major conformational shift could be explained by interruption of important tertiary contacts within the active site network. The induced alterations in structure and dynamics were not compensated by a scaffold responsible for structural stability of catalytic acid, resulting in compromised conformational gating. On the contrary, the same regions in S122A mutant were not affected substantially, suggesting that induced alterations were compensated by the scaffold, supporting structural stability of the nucleophile. In accord with NMR and HDX-MS data, metadynamics simulations also revealed that substitution of residues in the catalytic triad may cause alterations in delicate conformational equilibrium to a different extent, increasing the population of closed conformations. In particular, the metadynamics simulations predicted a major conformational shift in equilibrium from open states for sol-hMGL to closed states for D239A, which is in excellent agreement with the experimental results. Multiple lines of experimental evidence including NMR and HDX-MS data, and metadynamics simulations confirmed the critical role of the catalytic acid Asp-239 in the mediation of long-range communications regulating enzyme function.

In summary, we have found that substitution of catalytic triad residues as well as their proximal hydrogen bonded and aromatic neighbors alters the native conformational equilibrium of hMGL, increasing the relative population of closed (inactive) conformers. The overall robustness of hMGL communication networks is demonstrated by the subtle effects of point mutations on the conformational gating. However, removal or weakening of very critical allosteric links induced substantial conformational shift and significantly affected conformational gating. This study has revealed that catalytic acid substitution D239A caused significant modulation of structural networks for intraprotein communications. It appears that many distal contiguous regions of the enzyme substantially increased their dynamical flexibility, suggesting a major conformational shift toward more dynamical states and destabilization of the open conformers. Remarkably, the presence of a strong binder significantly shifts the equilibrium back to open conformers, allowing ligand entrance to the active site. Thus, conformational gating substantially compromised by nonconservative substitution of catalytic acid to alanine could be partially restored to its functional value. This result provides a strong indication for the existence of a robust scaffold around the catalytic acid of hMGL that provides the necessary structural support and is capable of compensating for the lost functionality of Asp-239 by maintaining its own functionality.

On the whole, it is reasonable to conclude that catalytic triad region of hMGL is comprised of structurally stable and highly interconnected residues preorganized into the integrated networks of tertiary communications via specific residue-residue contacts as well as quaternary communications via rigid secondary structure elements for the robust and rapid propagation of allosteric signals from the active to distal sites. The catalytic acid of hMGL could be considered as an essential residue for the integration and transmission of information to other residues in addition to its well-known role to bring the catalytic His-269 into the proper orientation for activation of Ser-122. This study provides new insights into the structure and dynamics of hMGL, and into the mechanism of catalytic triad action and allosteric regulation of serine proteases activity, in general. The results shown here may facilitate the identification of new druggable protein–ligand binding sites in hMGL.

## Methods

### Site-directed mutagenesis and enzyme assays

All the mutants in current study were designed and constructed based on a previously described N-terminal His_6_-tagged human MGL enzyme containing two serine residues instead of two leucines (double mutant L169S/L176S) in the lid domain^35,37^. This construct, named sol-hMGL, helps to avoid the need of detergent and obviate enzyme severe aggregation/precipitation. Additional single point mutations were introduced into the sol-hMGL construct to obtain a set of substitutions within the catalytic triad of enzyme (see SI for details). Detailed protocol for the assessment of hMGL hydrolytic activity was previously reported^37^ (see also SI).

### CD and NMR spectroscopy

CD measurements were performed using a Jasko J-815 CD spectrometer equipped with a Peltier temperature controller and single cuvette holder. All 1D and 2D solution NMR spectra were recorded on a Bruker AVANCE II 700 MHz spectrometer equipped with a 5-mm inverse triple resonance probe (see SI for details).

### Mass spectrometry of sol-hMGL and mutants

Peptic peptides were generated by passing of enzyme through the pepsin column and identified from tandem MS (MS/MS) data using a Thermo LTQ Orbitrap Elite mass spectrometer (Thermo Fisher, San Jose, CA), similarly to previous publication^37^.

### HDX-MS analyses

For HDX-MS analyses, the sol-hMGL and hMGL mutant proteins stock solutions were diluted in H_2_O buffer (20 mM sodium phosphate, 150 mM sodium chloride, 2 mM TCEP at pH 7.4) to prepare a 5 µM final concentration and equilibrated at 1 °C. HDX was conducted on a HDX PAL robot (LEAP Technologies, Carrboro, NC). See SI for further details.

### Well-tempered metadynamics simulations

The crystal structure of hMGL (3HJU) first underwent Protein Preparation Wizard in the Schrodinger Software package (Schrodinger, Inc, New York, NY, USA). Further geometry optimization of the catalytic triad residues as well as the water molecules residing within 3 Å of the catalytic triad was carried out by QM/MM method using Qsite. Well-Tempered metadynamics simulations were conducted using the OPLS3e force field in the Desmond software package^42^ (see SI for details).

### Disclaimer

Certain commercial materials and equipment are identified in order to adequately specify experimental procedures. Such identifications neither imply recommendation or endorsement by the National Institute of Standards and Technology nor do these imply that the material or equipment identified is the best available for the purpose.

## Supplementary information

Supplementary Information.

## Data Availability

All data generated or analyzed during this study are included in this published article (and its Supplementary Information file).
