# Supplementary material for: Conformational gating, dynamics and allostery in human monoacylglycerol lipase
Source: Sci Rep. 2020 Oct 28;10:18531. doi: 10.1038/s41598-020-75497-5 (PMC7595040; doi:10.1038/s41598-020-75497-5)
Supplement: Supplementary file 1 — Supplementary Information. [file 41598_2020_75497_MOESM1_ESM.pdf]

Supplementary Information for

Conformational Gating, Dynamics and Allostery in Human Monoacylglycerol Lipase

Sergiy Tyukhtenko[1*], Xiaoyu Ma[1], Girija Rajarshi[1], Ioannis Karageorgos[2,3], Kyle W. Anderson[2,3], Jeffrey W. Hudgens[2,3], Jason Guo[1,4], Mahmoud L. Nasr[5,6], Nikolai Zvonok[1], Kiran Vemuri[1], Gerhard Wagner[5], and Alexandros Makriyannis[1*]

[1]Center for Drug Discovery and Departments of Pharmaceutical Sciences and Chemistry and Chemical Biology, Northeastern University, Boston, Massachusetts 02115-5000

[2]BioProcess Measurements Group, Biomolecular Measurement Division, National Institute of Standards & Technology, Rockville, MD 20850.

[3]Institute for Bioscience and Biotechnology Research, 9600 Gudelsky Drive, Rockville, MD 20850.

[4]Barnett Institute for Chemical and Biological Analysis, Northeastern University, Boston, Massachusetts 02115-5000.

[5]Department of Biological Chemistry and Molecular Pharmacology, Harvard Medical School, Boston, Massachusetts, 02115.

[6]Department of Medicine, Renal Division and Division of Engineering in Medicine, Brigham and Women's Hospital, Harvard Medical School, Boston, Massachusetts, 02115.

[*]To whom correspondence should be addressed: s.tyukhtenko@northeastern.edu, a.makriyannis@northeastern.edu

SUPPORTING RESULTS

Evaluation of the Effect of Active Site Residue Mutation on the Stability of hMGL Fold. The CD spectra recorded in the far-UV region (190-260 nm) provide evidence that there is no sign of a significant amount of random coil structures (Fig. S1). Thermal unfolding curves for these mutants were obtained by monitoring changes in the negative CD signal at 222 nm as a function of temperature (Fig. S2). All mutants have melting temperatures similar or slightly higher compared to sol-hMGL ($T_{m\ \mathrm{sol\text{-}hMGL}}$ = 60.9 ± 0.6 ^{o}C (1σ))[1]. Secondary structure and the tertiary fold of constructs were also evaluated using 2D ^{1}H -^{15}N heteronuclear single quantum coherence (HSQC) NMR experiments (Fig. S3). The crosspeaks are well dispersed, indicating the presence of distinct hMGL secondary structures and stable tertiary folds. For D239A mutant the dispersion of the peaks looks smaller, and at first glance, may reflects partial unfolding of the enzyme. However, the melting curve does not suggest unfolding upon D239A substitution (Fig. S2). The observed extreme broadening, heterogeneity and smaller dispersion of resonance lines in this case may be most explained by the increased dynamic flexibility.

Global Conformational and Dynamic Changes upon Ligand Binding. 2D ^{1}H -^{15}N HSQC NMR spectra provide important information on the secondary structure and tertiary fold of hMGL constructs and their complexes with ligand. Our previous NMR studies revealed that hMGL constructs demonstrate broadened lines in the HSQC spectra[2, 1] as a result of stochastic equilibria between different conformational states occurring on the microsecond to millisecond time scale. Therefore, it was not feasible to proceed with NMR sequential assignments, due to overlapping and significant reduction in the sensitivity of signals arising from severe line broadening caused by conformational heterogeneity.

Here, we have observed similar conformational heterogeneity for the catalytic triad mutants. Superimposed HSQC spectra of free and bound states for these mutants are presented in Fig. S4. All spectra for free states contain the subsets of relatively well dispersed crosspeaks in addition to a large number of unresolved resonances. This behavior is characteristic of intermediate to slow exchange between multiple

hMGL conformations. In the case of D239A (Fig. S4c), the extreme peak shape heterogeneity and overlapping peaks indicates a state of substantially increased dynamic flexibility. Nevertheless, the peaks for all mutants are distributed enough across the spectra, giving an overall view of the enzyme's behavior during ligand binding. Remarkably, many resonance lines in the bound states with compound 1 appear to sharpen significantly, indicating more homogeneous dynamics in the bound states.

METHODS

Site-directed Mutagenesis, Expression and Purification of sol-hMGL and Mutants. The full DNA sequence for designed substitutions was submitted to GenScript (Piscataway, NJ) and synthesized DNA were provided upon full sequencing and cloning in pET-45b(+). Each mutated plasmid was transformed and expressed in BL21 (DE3) *E.coli* cells. Ten different constructs were generated based on this sol-hMGL template: S122A, S122C, S122T, H269A, D239A, D239N, H121A, L241A, C242A and Y268A. Recombinant enzyme expression and purification were performed based on a previously reported protocol[2]. ^{15}N-labeled cells for the catalytic triad mutants were grown in Spectra-9 media for bacterial cell growth (Cambridge Isotopes Labs) and purified. The expression levels and yields were comparable to those of sol-hMGL. SDS-PAGE and size-exclusion chromatography were employed to confirm the purity and oligomeric state of the protein preparations (data not shown). All mutants were high purity and were found to exist in solution as mixtures of monomers and oligomers.

Enzyme Assays. The effects of mutations on enzyme hydrolytic activity were determined by incubation of purified construct with endogenous substrate 2-arachidonoylglycerol (2-AG) and quantifying the product arachidonic acid (AA) by high-performance liquid chromatography (HPLC). The 2-AG substrate was generously supplied by NIH (Bethesda, MD) and AA was purchased from Nu-Chek Prep (Elysian, MN). The initial velocity data generated from original UV-Vis and HPLC results were fitted to the Michaelis-

Menten equation plot using nonlinear regression in GraphPad Prism 5.0 (San-Diego, CA) with the aim to estimate V_{max}, K_m, k_{cat} and catalytic efficiency (k_{cat}/K_m).

Circular Dichroism Spectroscopy. Potential conformational changes in the secondary structure of mutants were monitored in the far-UV region between 190 and 260 nm with enzymes concentrations 10 μM (300 μL) in a quartz cuvette with a path length of 1 mm. The components of buffer (pH 7.4) for CD experiments were 20 mM sodium phosphate, 100 mM NaCl and 2 mM TCEP. Three accumulations of scans were taken and averaged to get the complete spectra.

Nuclear Magnetic Resonance Spectroscopy and Data Analysis. For 1D ^{1}H NMR spectra, 3-9-19 WATERGATE[3] pulse sequence (p3919fpgp) with gradients and additional flip back pulse was used for optimal detection of downfield exchangeable proton resonances. Exponential multiplication (broadening factor lb = 20 Hz) was applied for all 1D NMR spectra. 2D ^{1}H -^{15}N HSQC NMR spectra were acquired using the standard Bruker pulse sequences supplied with AVANCE 700 spectrometer, as detailed before[2]. The data were processed and visualized using Topspin 3.2 (Bruker). Enzyme samples prepared for NMR experiments were 0.1-0.3 mM in 95% H_2O, 5% D_2O buffer containing 20 mM sodium phosphate 100 mM NaCl and 2 to 5 mM TCEP (tris(2-carboxyethyl)phosphine) at pH7.4. Sample volume was 0.5-0.6 mL. Chemical shifts were referenced to an internal DSS (sodium 2,2-dimethyl-2-silapentane-5-sulfonate) standard (20 μM). Ligand binding experiments were performed using a 50 mM stock solution of compound 1 dissolved in DMSO-d_6. Compound 1[4] was synthesized at the Center for Drug Discovery.

HDX-MS and HDX Data Processing. Protein solutions (5 µL) were diluted into 25 µL D_2O buffer (20 mM sodium phosphate, 150 mM sodium chloride, 2 mM TCEP at pD 7.4) at 25 °C. At selected times (0 s, 30 s, 5 min, 15 min, 1 h, and 4 h) the HDX sample was quenched by mixing with 35 µL quench buffer (3 M urea, 0.1 M sodium phosphate at pH 2.5) at 1 °C. The quenched solution was injected into an on-line

immobilized pepsin column for 3 min. The digested protein solution was trapped on a C18 guard column (1.0 mm diameter x 5 mm length, 5 µm; Grace Discovery Sciences, Deerfield, IL) and separated with a C18 analytical column (1.0 mm diameter x 5 mm length, 1.9 µm, Hypersil GOLD, Thermo Scientific) via a Thermo Fisher Ultimate 3000 UPLC with a 9.5 min gradient operated with a binary mixture of solvents A and B at 50 µL/min flow rate. The gradient settings used were: 5 % to 35 % solvent B for 3 min, 35 % to 60 % solvent B for 5 min, 60 % to 100 % solvent B for 0.5 min, isocratic flow at 100 % solvent B for 0.5 min, and a return in 5 % solvent B for 0.5 min. Solvent A was water containing 0.1 % formic acid and solvent B was 80 % acetonitrile and 20 % water containing 0.1 % formic acid. LC connection lines and valves were housed in a refrigerated compartment at 2 °C. Peptides were mass analyzed on a Thermo Orbitrap Elite (Thermo Fisher, San Jose, CA). The instrument settings were: spray voltage, 3.7 kV; sheath gas flow rate, 25 (arbitrary units); capillary temperature, 275 °C. In the Orbitrap stage, MS spectra were acquired with the resolution set at 60000. Three replicates for each ion-exchange time point were obtained. From mass spectra obtained during HDX-MS experiments, the centroid of each deuterated peptide envelope and the relative deuterium uptake by each peptide were calculated by HDX WorkBench[5]. Corrections for back exchange were made by considering the values of 80 % deuterium content of the exchange buffer and an estimated 70 % deuterium recovery.

Well-Tempered metadynamics simulations. Briefly, the catalytic triad residues and adjacent water molecules were set as the QM region at B3LYP/6-31G (d, p) level, whereas the rest of the protein was set as the MM region. The Qsite calculations were conducted under gas phase, where QM region was optimized up to 700 steps and the MM region remained unchanged. The sol-hMGL as well as each of the mutants was first solvated in SPC water with 10 Å buffer and 0.15 M NaCl. The systems were then equilibrated using the default protein relaxation protocol first with restraints on the solute heavy atoms: 1) 100 ps of Brownian dynamics NVT at 10 K, 2) 12 ps of NVT simulation at 10K, 3) 12 ps of NPT simulation at 10K, and 4) 12 ps of NPT simulation at 300K; followed by 24 ps of NPT simulations at 300K without any restraints. The well-tempered metadynamics was carried out for 350 ns at 300K using a Gaussian width of 0.1 Å with the

following two collective variables: *CV1* (distance between the centers of mass of 176–179 Cα and 150–157 Cα) and *CV2* (distance between mass centers of 176–179 Cα and 158–164 Cα). The initial Gaussian height was set at 0.03 kcal/mol, and was gradually decreased on the basis of adaptive bias with a *ΔT* of 1200 K.

REFERENCES

1 Tyukhtenko, S. *et al.* Effects of Distal Mutations on the Structure, Dynamics and Catalysis of Human Monoacylglycerol Lipase. *Scientific Reports* **8**, doi:10.1038/s41598-017-19135-7 (2018).

2 Tyukhtenko, S. *et al.* Specific Inter-residue Interactions as Determinants of Human Monoacylglycerol Lipase Catalytic Competency A ROLE FOR GLOBAL CONFORMATIONAL CHANGES. *J. Biol. Chem.* **291**, 2556-2565, doi:10.1074/jbc.M115.670257 (2016).

3 Sklenar, V., Piotto, M., Leppik, R. & Saudek, V. Gradient-Tailored Water Suppression for 1H-15N HSQC Experiments Optimized to Retain Full Sensitivity. *Journal of Magnetic Resonance, Series A* **102**, 241-245, doi:10.1006/jmra.1993.1098 (1993). (1993).

4 Chevalier, K. *et al.* Heteroaromatic and aromatic piperazinyl azetidinyl amides as monoacylglycerol lipase inhibitors. USA patent 8,415,341 (2013).

5 Pascal, B. D. *et al.* HDX Workbench: Software for the Analysis of H/D Exchange MS Data. **23**, 1512-1521, doi:10.1007/s13361-012-0419-6 (2012).

Figure S1. Comparison of the far-UV CD spectra for the catalytic triad mutants of sol-hMGL. The protein concentrations were ~ 10 μM.

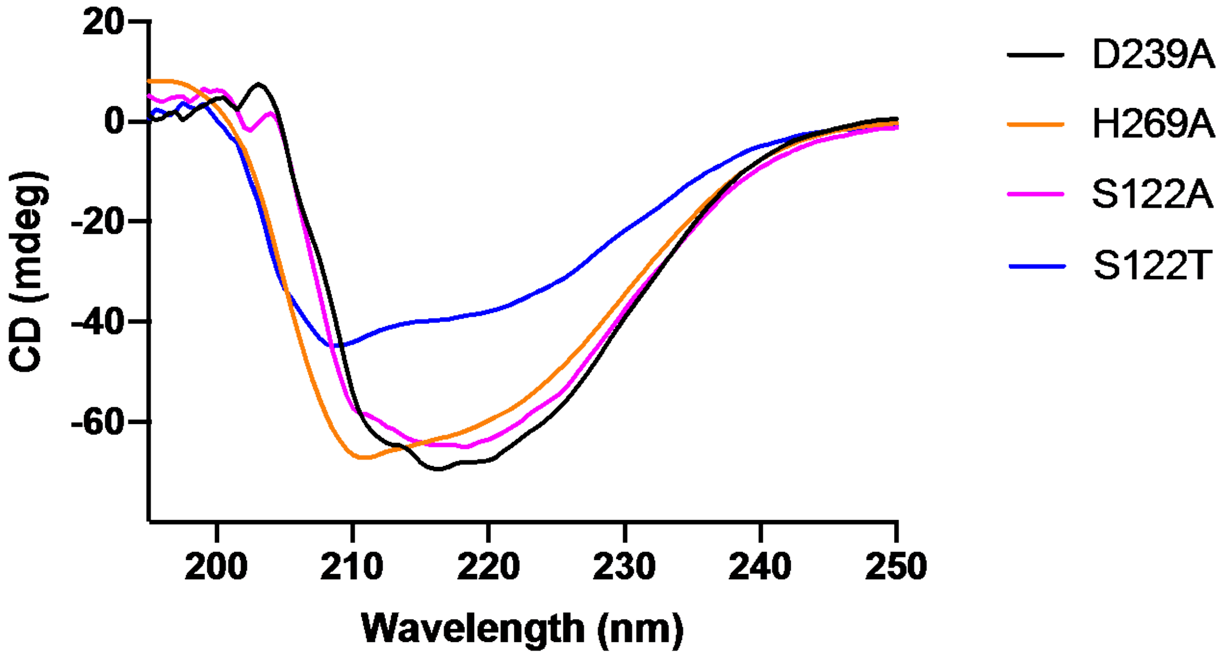

Figure S2. Thermal denaturation curves for the catalytic triad mutants of sol-hMGL. Enzymes were subjected to a temperature gradient in 20 mM sodium phosphate, 100 mM NaCl, 2 mM TCEP buffer, pH 7.4, and unfolding was followed by monitoring the CD signal at 222 nm. Data points are shown in black circles and the sigmoidal fits used to determine T_m.

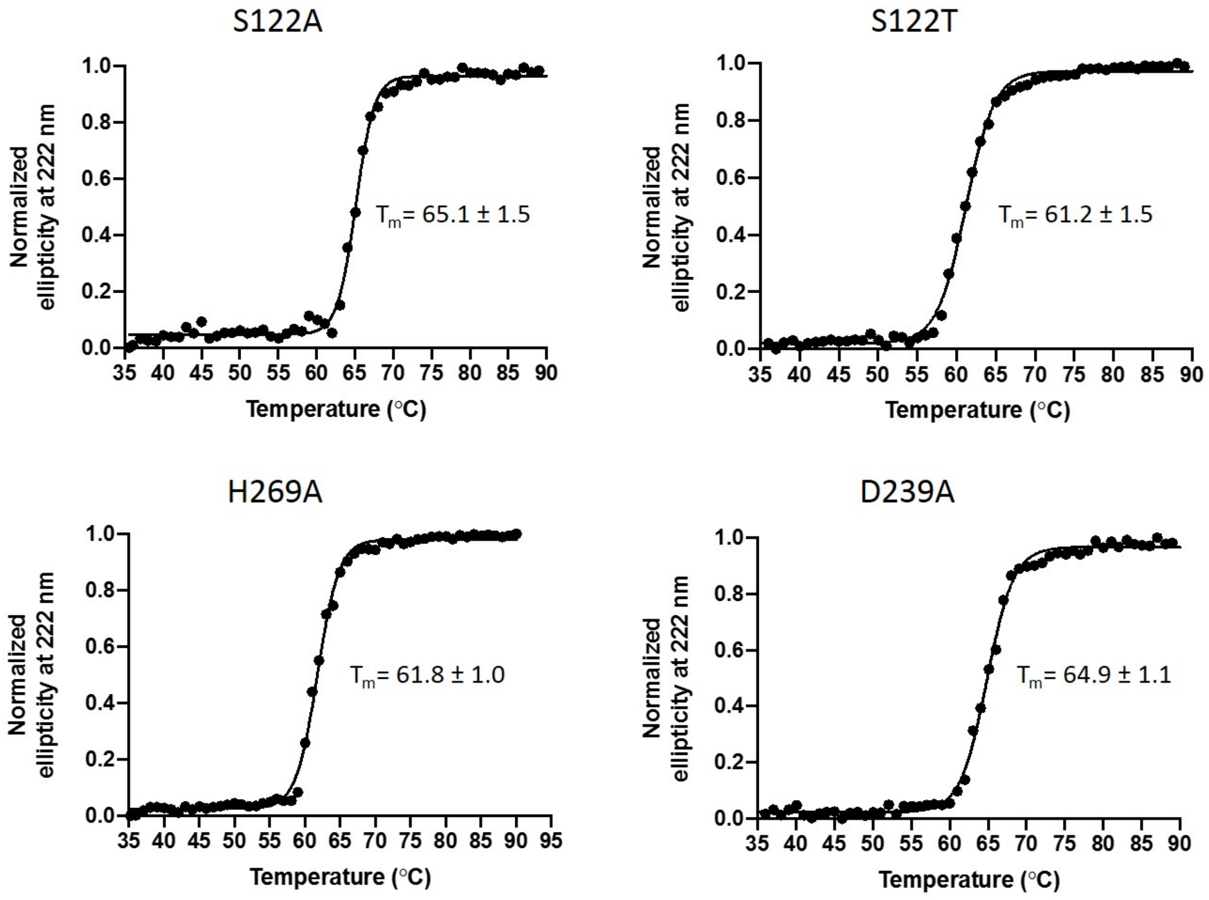

Figure S3. Superimposition of 2D ^{1}H -^{15}N HSQC spectra of uniformly ^{15}N-labeled enzymes in free (red) and bound with compound 1 (blue) forms. (a) S122A mutant, (b) H269A mutant, and (c) D239A mutant at 310 K, pH 7.4.

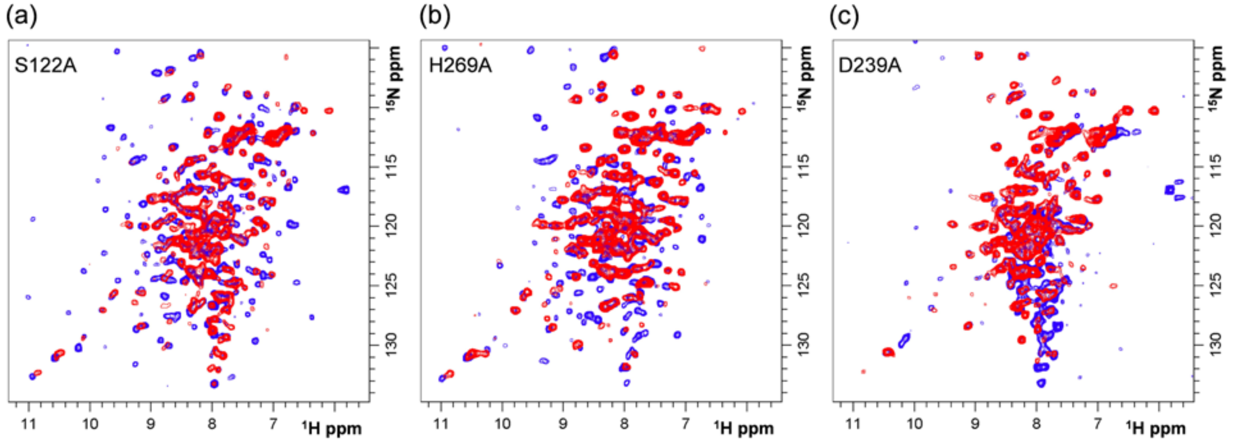

Figure S4. Reaction curves of initial velocity versus 2-AG concentration for sol-hMGL and mutants fitted to the Michaelis-Menten equation.

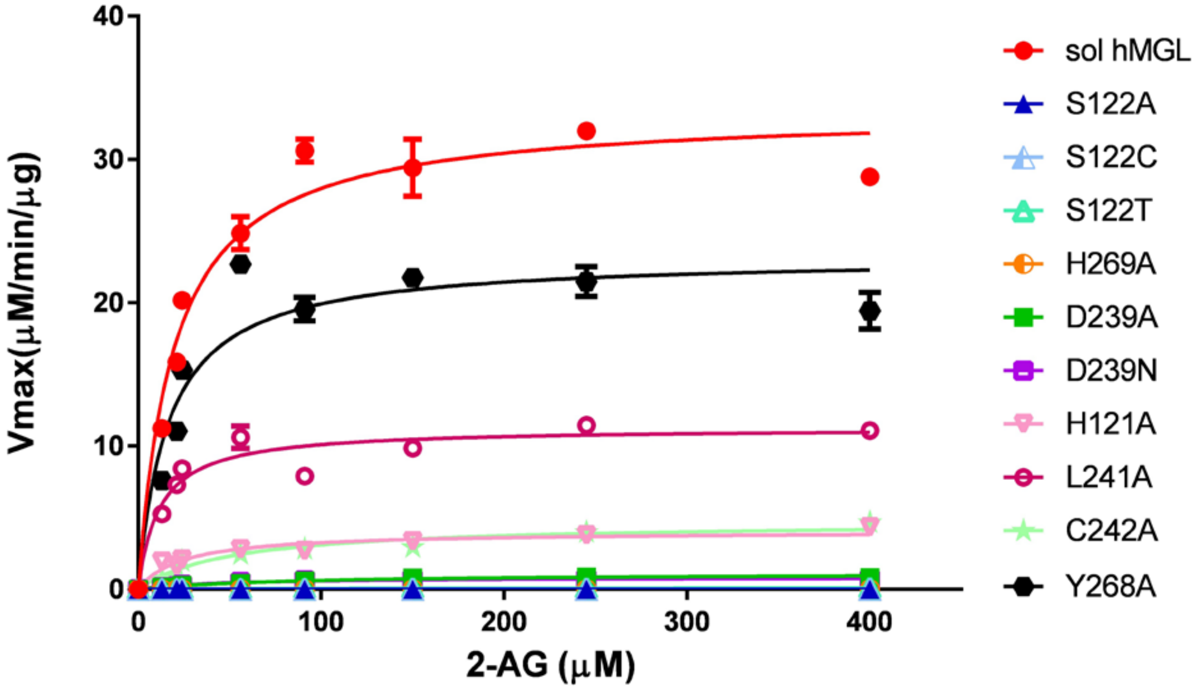

Figure S5. The downfield ^{1}H NMR spectra of S122C mutant showing the temperature dependence. The ^{1}H resonance signal at 18 ppm, corresponding to the $H^{\delta 1}$ proton of doubly protonated (positively charged) catalytic His-269, becomes detectable only at low temperatures due to its slower exchange rate with solvent.

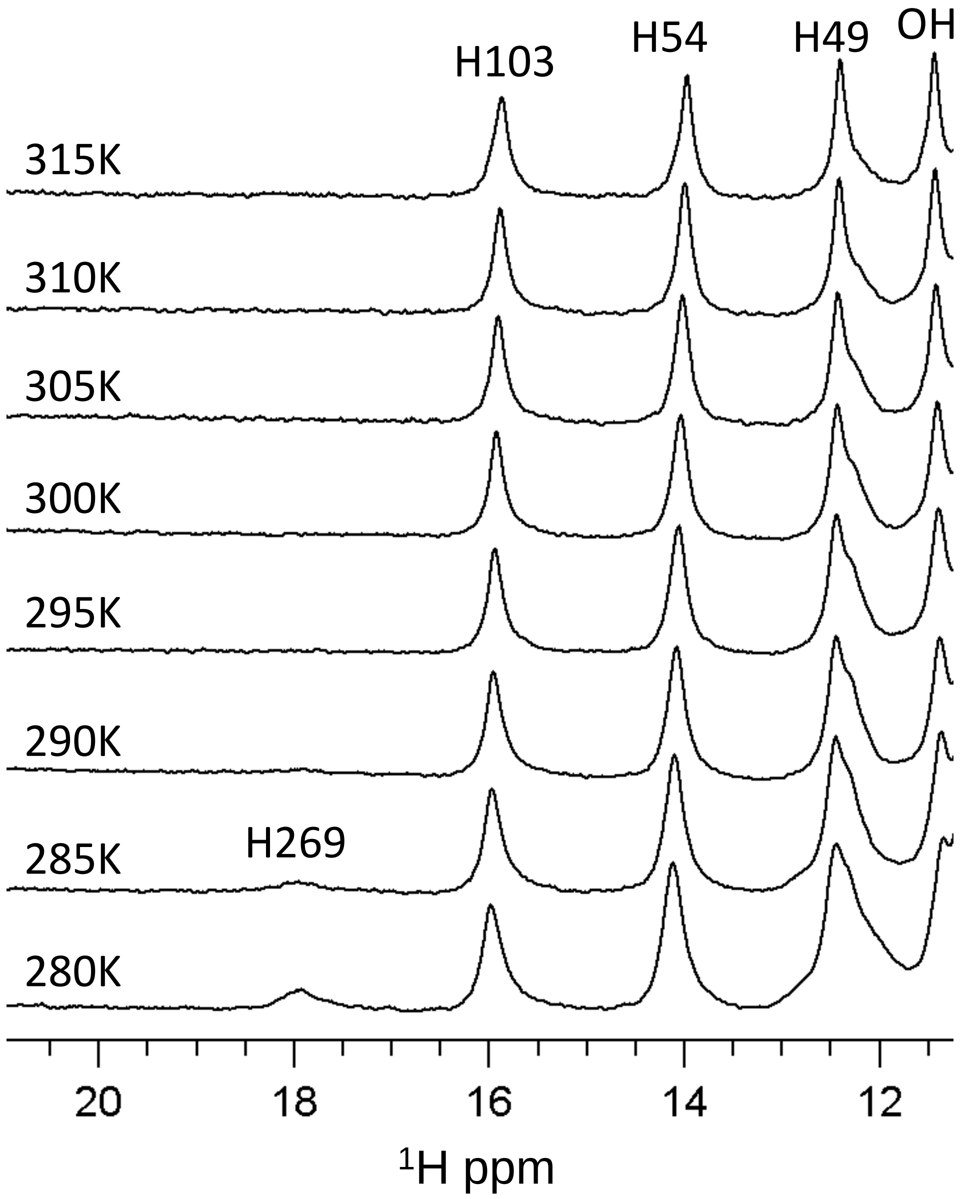

Figure S6. Sequence coverage map for peptic peptides that were identified by MS/MS spectra for sol-hMGL. The peptides are presented as bars.

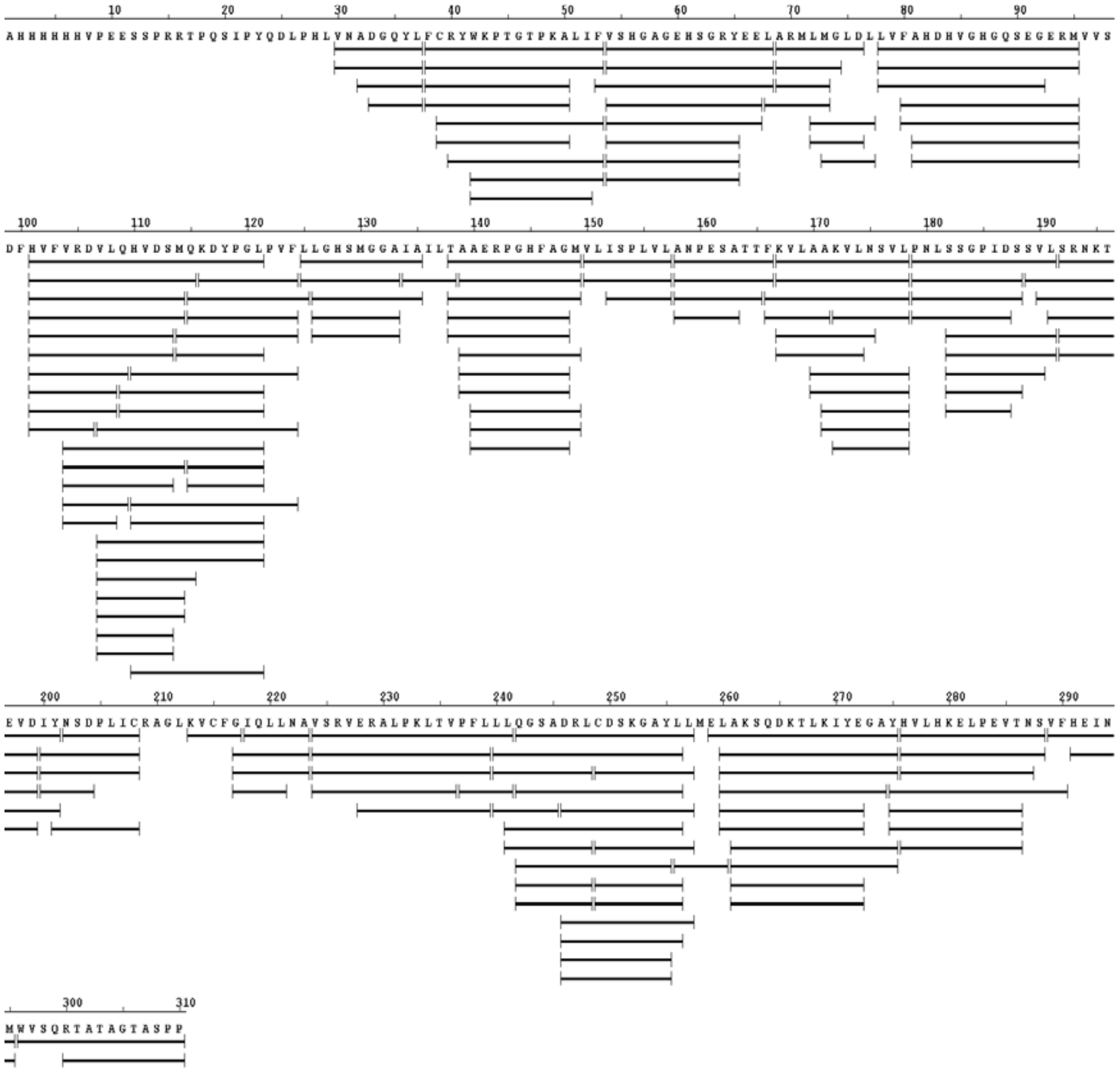

sol-hMGL

Figure S7. Sequence coverage map for peptic peptides that were identified by MS/MS spectra for S122A mutant. The peptides are presented as bars.

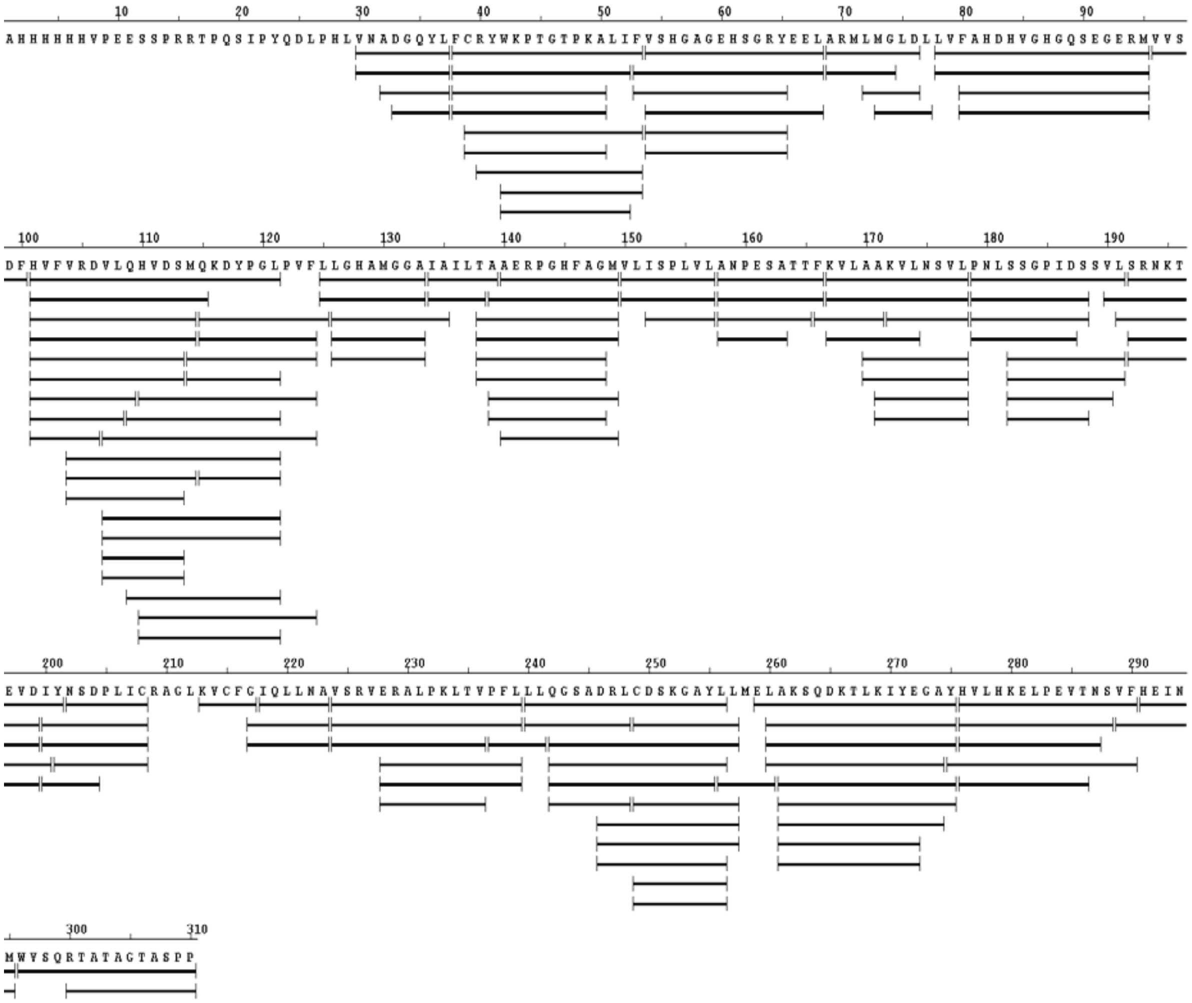

S122A

Figure S8. Sequence coverage map for peptic peptides that were identified by MS/MS spectra for D239A mutant. The peptides are presented as bars.

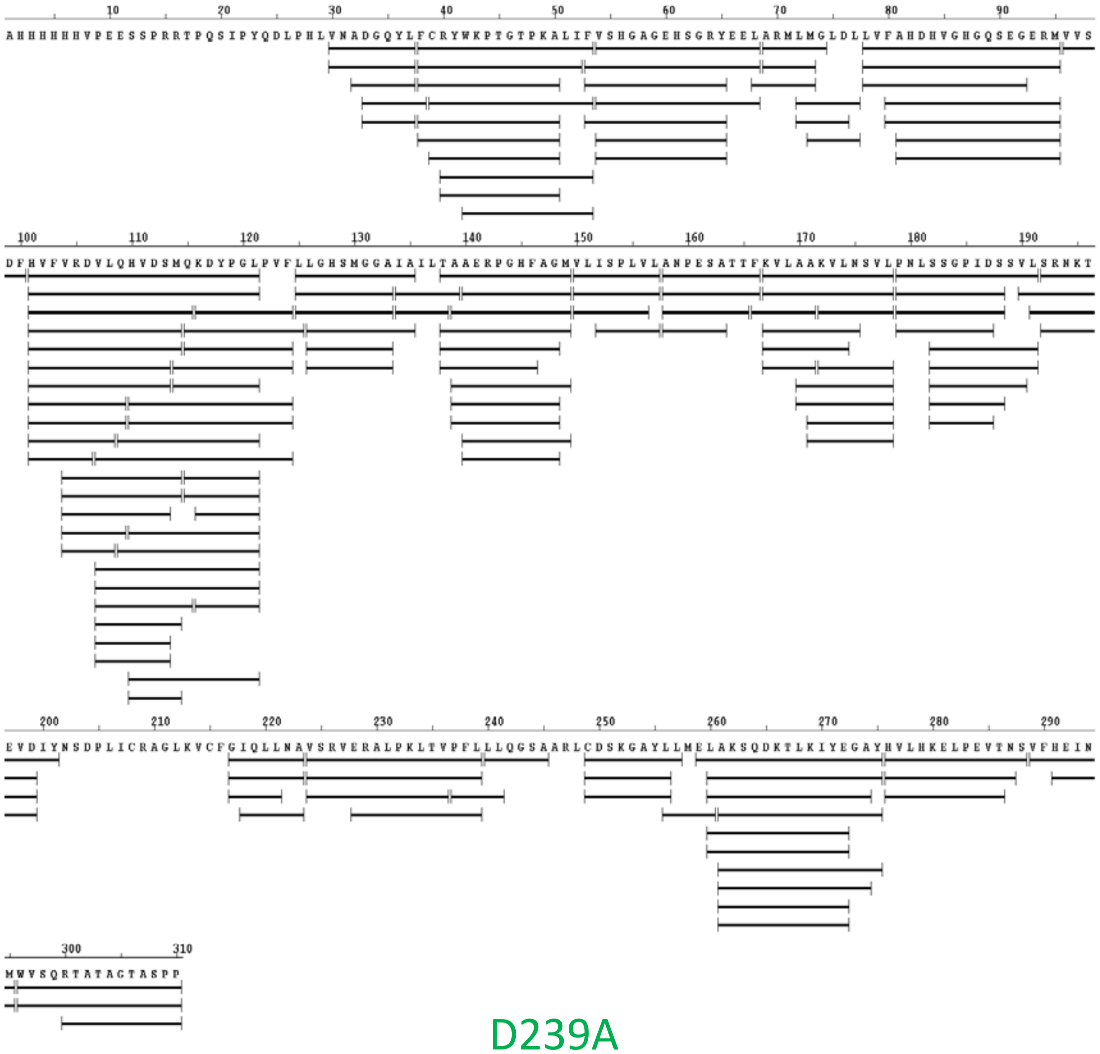

D239A

Table S1. Percentage deuterium uptake for the peptides detected in sol-hMGL and two catalytic triad mutants S122A and D239A for D_2O immersion of t = 30 s to t = 4 h. The data points are presented as mean ± standard error (1σ), which were computed from data observed in the triplicate experiments. The values are adjusted for back exchange.

		30sec			5min			15min			1h			4h		
Peptide	Sequence	sol-hMGL	S122A	D239A	sol-hMGL	S122A	D239A	sol-hMGL	S122A	D239A	sol-hMGL	S122A	D239A	sol-hMGL	S122A	D239A
ADGQYL	25-30	14±0.6	12.2±0.7	19.0±1.2	18±0.9	13.6±0.9	21.9±1.2	17.3±1.1	16.6±0.4	23.7±2.4	22.6±1.0	20.9±0.3	33.2±1.8	31.5±1.0	30.3±1.1	49.8±0.1
FCRYWKPTGTPKA	31-43	32.9±0.8	33.1±0.0	40.3±0.1	43.4±0.3	42.8±0.5	48.5±0.6	47.2±1.1	47.2±0.4	49.8±0.3	51.1±0.9	50.4±0.6	49.0±2.5	51.8±1.3	51.2±0.7	49.9±0.9
WKPTGTPKALIF	35-46	25.2±0.7	25.8±0.5	40.2±2.6	28.3±0.3	27.9±0.7	37.0±0.1	31.0±0.7	31.8±0.6	38.7±0.8	34.6±0.7	34.7±0.8	45.2±0.9	35.7±0.8	36.3±1.1	44.9±1.9
FVSHGAGEHSGRYEEL	46-61	21.9±0.1	20.6±0.3	24.5±0.5	28.1±0.2	26.2±0.2	34.8±0.6	29.4±0.6	27.8±0.3	32.0±1.7	31.7±0.1	29.4±0.8	35.4±0.4	32.4±0.2	31.7±0.7	32.6±0.7
ARMLMG	62-67	17.3±0.9	13.6±0.3	23.5±0.1	23.6±0.2	21.7±0.5	32.2±0.8	24.0±0.4	22.8±0.0	32.6±0.0	25.9±0.4	24.7±0.3	29.6±1.2	28.9±0.5	27.2±1.1	35.9±0.4
LVFAHDHVGHGQSEGERM	71-88	18.3±0.3	17.8±0.5	22.6±0.4	21.4±0.4	20.1±0.4	27.3±1.0	21.8±0.3	21.2±0.5	30.3±0.3	24.1±0.5	22.5±0.4	38.6±0.8	25.0±0.4	24.8±0.7	48.9±0.7
HVFVRDVLQHVDS	94-106	8.8±0.1	8.2±0.5	12.0±0.7	10.8±0.4	9.9±0.2	15.7±0.3	10.5±0.3	11.0±0.7	18.0±1.5	12.7±0.5	12.9±0.6	22.3±0.8	14.7±0.4	16.4±0.6	33.2±0.2
QHVDSMQKDYPGL	102-114	14.0±0.3	14.1±0.4	24.0±0.5	22.8±0.4	22.0±0.3	30.4±0.8	25.5±0.3	25.7±0.3	32.6±0.1	30.1±0.1	28.8±0.3	38.6±1.1	33.9±0.4	33.8±0.6	43.4±0.9
LLGHSMGGA	118-126	15.4±0.2	18.5±0.5	19.5±0.8	16.6±0.4	19.2±0.3	22.0±0.5	16.1±0.5	20.5±0.3	23.0±0.6	18.0±0.5	21.1±0.3	26.1±0.6	17.9±0.8	23.0±0.4	29.0±0.7
IAILT	127-131	6.4±0.4	5.5±0.3	11.5±0.6	7.9±0.7	6.6±0.2	14.7±0.7	7.4±0.3	7.4±0.8	16.7±1.2	8.8±1.1	8.7±1.1	23.2±0.5	8.3±1.2	9.1±0.6	38.4±0.0
AAERPGHFAGM	132-142	19.8±0.3	19.4±0.4	24.4±0.8	23.6±0.4	22.4±0.5	25.7±0.8	25.9±0.6	26.2±0.2	30.2±1.4	34.6±0.7	33.2±0.1	34.3±1.2	40.1±0.9	40.0±0.7	38.0±1.0
VLISPLVL	143-150	10.1±0.2	12.0±0.3	43.6±0.5	28.0±0.9	24.4±0.8	67.3±0.5	39.4±0.6	33.5±0.2	72.1±0.6	55.6±0.7	48.0±1.1	75.0±0.8	64.9±0.8	60.5±0.8	80.8±0.9
ANPESATTF	151-159	75.9±0.7	74.8±0.5	75.7±1.1	77.1±0.6	75.3±0.3	77.9±0.3	78.2±0.3	76.2±0.5	78.0±0.3	77.0±0.8	74.8±1.2	76.6±0.6	76.5±0.8	77.1±0.0	77.2±0.9
KVLAAKVLNSVL	160-171	73.1±0.3	65.9±0.2	52.6±0.3	75.8±0.2	70.7±0.6	55.5±0.1	76.9±1.1	73.3±0.3	61.3±0.6	77.6±0.1	75.1±0.3	64.9±0.8	78.3±0.4	77.2±0.9	68.6±1.2
PNLSSGPIDSSVL	172-184	68.6±0.5	67.1±0.1	70.5±0.8	71.1±0.5	69.4±0.2	72.4±0.3	72.3±0.5	70.7±0.5	72.4±0.0	71.8±0.3	69.9±0.9	69.6±0.7	71.2±0.7	72.1±0.5	70.9±0.5
SRNKTEVD	185-192	44.5±0.7	38.8±0.4	42.4±0.8	46.3±0.5	42.1±0.3	42.6±0.9	47.9±0.9	44.1±0.4	46.1±0.3	47.8±0.5	43.8±0.1	44.3±0.2	49.2±0.3	46.8±0.5	45.8±0.6
GIQLLNA	210-216	58.0±0.3	49.9±0.5	61.0±0.3	78.0±0.4	70.9±0.6	71.2±0.7	79.9±0.1	73.7±1.1	72.1±1.7	82.0±0.3	76.6±1.0	70.8±1.3	82.0±1.4	80.7±1.4	73.6±0.8
VSRVERALPKLTVPFL	217-232	23.2±0.4	22.2±0.2	31.7±0.6	38.4±0.6	34.7±0.3	46.5±0.4	44.2±1.0	40.4±0.0	50.6±1.4	51.0±0.9	46.1±0.8	54.0±1.3	56.5±1.0	53.5±0.6	58.6±1.7
PFLLL	230-234	2.5±0.3	3.0±0.4	10.2±0.9	5.4±1.0	4.9±1.1	17.1±1	4.7±0.3	5.8±0.9	15.3±3.2	5.3±1.3	5.2±0.6	20.7±3.3	5.9±1.0	5.1±2.3	19.5±2.7
CDSKGAYL	242-249	32.1±0.5	28.6±0.4	47.8±0.7	49±0.3	42.9±0.5	62.9±0.3	53.9±0.8	48.7±0.8	66.6±1.1	56.5±0.8	51.0±0.4	65.5±1.1	56.6±1.1	53.8±1.1	73.1±2.8
LAKSQDKTLKIYEGAY	253-268	30.9±0.4	30.0±0.3	34.8±0.3	44.0±0.0	41.0±0.3	45.1±0.2	46.1±0.8	43.7±0.3	46.5±1.4	48.8±0.6	45.4±0.1	48.1±1.1	51.5±0.4	49.4±0.6	52.8±0.2
HVLHKELPEVTNS	269-281	40.0±0.1	37.1±0.3	51.6±0.4	51.2±0.6	47.7±0.6	51.8±0.7	51.9±1.1	49.7±0.5	52.9±0.6	53.3±0.4	50.9±0.3	52.3±1.1	53.3±0.6	53.1±0.6	53.1±1.3
WVSQRTATAGTASPP	289-303	62.7±0.5	62.5±0.4	71.8±1.8	69.8±0.4	68.4±0.3	78.1±1.4	73.6±0.4	71.8±0.5	76.4±0.5	74.5±0.8	73.3±0.1	72.9±1.4	74.4±0.8	76.0±0.2	74.9±1.0
